# mRNA delivery of a class 1/4 SARS-CoV-2 neutralizing antibody protects against diverse sarbecoviruses in a lethal mouse challenge model

**DOI:** 10.1073/pnas.2536870123

**Published:** 2026-06-08

**Authors:** Ashwin N. Skelly, Chengcheng Fan, Jennifer R. Keeffe, Arya Ökten, Edem Gavor, Maddy L. Newby, Joel D. Allen, Edward F. Kreider, Wenge Ding, Rebecca A. Osbaldeston, Younghoon Park, Andrew J. Connell, Melinda G. Lituchy, Frederic Bibollet-Ruche, Katelyn M. Radford, Anthony P. West, Mario A. Peña-Hernández, Kendra Cruickshank, Weimin Liu, Yingying Li, Amie Albertus, Brieyanna McWilliams, Ronnie M. Russell, Kylie M. Konrath, Jonathan L. Torres, Meng Yuan, Hongmei Gao, David C. Montefiori, Michael S. Saag, Paul A. Goepfert, Daniel W. Kulp, Andrew B. Ward, Ian A. Wilson, George M. Shaw, Raiees Andrabi, Max Crispin, Drew Weissman, Craig B. Wilen, Pamela J. Bjorkman, Beatrice H. Hahn

**Affiliations:** a Department of Medicine, University of Pennsylvania; Philadelphia, PA 19104, USA.; b Department of Microbiology, University of Pennsylvania; Philadelphia, PA 19104, USA.; c Division of Biology and Biological Engineering, California Institute of Technology; Pasadena, CA 91125, USA.; d Department of Laboratory Medicine, Yale School of Medicine; New Haven, CT 06510, USA.; e Department of Immunobiology, Yale School of Medicine; New Haven, CT 06510, USA.; f School of Biological Sciences, University of Southampton; Southampton, SO17 1BJ, UK.; g Vaccine and Immunotherapy Center, The Wistar Institute; Philadelphia, PA 19104, USA.; h Department of Integrative Structural and Computational Biology, The Scripps Research Institute; La Jolla, CA 92037, USA.; i Duke Human Vaccine Institute, Duke University Medical Center; Durham, NC 27710, USA.; j Department of Surgery, Duke University Medical Center; Durham, NC 27710, USA.; k Department of Medicine, University of Alabama at Birmingham; Birmingham, AL 35233, USA.; l Department of Microbiology, University of Alabama at Birmingham; Birmingham, AL 35233, USA.; m O’Neal Comprehensive Cancer Center, University of Alabama at Birmingham; Birmingham, AL 35233, USA.

**Keywords:** Biological Sciences, Microbiology, sarbecovirus, class 1/4 anti-RBD broadly neutralizing antibody, mRNA antibody delivery, passive immunization, hybrid immunity

## Abstract

The unyielding antigenic drift of severe acute respiratory syndrome coronavirus 2 (SARS-CoV-2), as well as the threat of future zoonotic sarbecovirus spillovers, has prompted the search for broadly neutralizing antibodies (bNAbs) to inform rational therapeutic and vaccine design. Here, we isolated and characterized 20 receptor binding domain (RBD)-directed bNAb lineages from a serially-sampled SARS-CoV-2 patient who was infected and vaccinated during the early months of the pandemic. Thirteen of these targeted the highly conserved, cryptic class 1/4 or class 4 RBD epitopes and had long (18–26 amino acid) heavy chain complementarity determining region 3 (CDRH3) loops that utilized the IGHD3–22 gene segment. Five bNAbs potently neutralized all 18 viruses in a panel containing SARS-CoV-2 variants up to the recently emerged XBB.1.5 and JN.1 strains as well as diverse sarbecoviruses from other clades. Structural analyses of the Ab401 and Ab568 bNAbs complexed with RBD and Spike trimer, respectively, revealed recognition features in common with other class 1/4 bNAbs. Prophylactic administration of Ab401 as a recombinant protein afforded robust protection against infectious challenge with either SARS-CoV-2_WA1 or a related bat sarbecovirus with zoonotic potential. A similar level of protection was achieved when the heavy and light chains of Ab401 were delivered as lipid nanoparticle-encapsulated mRNAs. These data expand the arsenal of SARS-CoV-2 bNAbs for clinical development and identify mRNA-based antibody delivery as a promising platform for both pandemic preparedness and protection of immunocompromised patients against emerging sarbecovirus variants.

## Introduction

The development and widespread deployment of vaccines against severe acute respiratory syndrome coronavirus 2 (SARS-CoV-2) have substantially curtailed the coronavirus disease 2019 (COVID-19) pandemic. However, vaccine efficacy has been poor in individuals with congenital and acquired immunodeficiencies, leaving them at high risk for COVID-19 related morbidity and mortality (1). Monoclonal antibodies (mAbs) have been used both prophylactically and therapeutically in these immunocompromised patients, as well as in the treatment of severe COVID-19 in immunocompetent individuals (2, 3). Nevertheless, the unrelenting antigenic drift since the onset of the pandemic has given rise to resistant SARS-CoV-2 variants, rendering almost all clinically approved mAbs obsolete (4, 5). Indeed, while the United States FDA initially issued emergency use authorizations for several mAbs, they have since revoked all but one of these authorizations, citing poor efficacy against currently circulating strains (4–6).

The primary mechanism of antibody-mediated protection against SARS-CoV-2 is viral neutralization. Neutralizing antibodies (NAbs) target numerous epitopes on the SARS-CoV-2 Spike (S) protein, which comprises S1 and S2 subunits that mediate receptor recognition and cell membrane fusion, respectively (7, 8). NAbs primarily target the S1 receptor binding domain (RBD), and six distinct RBD epitope classes have been defined: class 1, 2, 3, 4, 5, and 1/4 (9–14). The most potent NAbs recognize class 1 and 2 epitopes, which overlap with the binding site of the human angiotensin-converting enzyme 2 (ACE2) host receptor (11–15). However, these sites are highly variable and are frequently mutated in SARS-CoV-2 variants of concern (VOCs) (12). As such, class 1 and 2 anti-RBD antibodies, including most previously licensed mAbs, have largely been unable to accommodate this antigenic diversity and thus exhibit poor efficacy against contemporary strains (16). In contrast, several NAbs targeting the more-conserved class 1/4, 3, 4, and 5 RBD epitopes, which are located on the proximal portion of RBD (adjacent to the other S1 subunit domains), have retained activity against recently emerged VOCs and even cross-neutralize other sarbecoviruses (17–23). Most of these broadly neutralizing antibodies (bNAbs) target cryptic epitopes outside the ACE2 binding site and frequently require conformational rearrangements in Spike to permit antibody binding. Although these bNAbs exhibit considerable breadth, their clinical utility is limited by their moderate-to-low potency. However, there are important exceptions, including class 1/4 antibodies that bind to the highly conserved class 4 RBD epitope but are oriented such that the intact antibody sterically occludes the class 1 epitope and ACE2 binding site. As such, class 1/4 bNAbs generally exhibit substantial breadth and potency and thus represent high-value targets for vaccine design as well as prophylactic and therapeutic mAb applications (24, 25).

The isolation and characterization of large panels of bNAbs, including those with the same epitope specificities, has been instrumental in advancing preventative and therapeutic strategies to combat viral infections. Studies of HIV-1 bNAbs, for example, have defined neutralization determinants that resist viral escape and have guided rational immunogen design efforts (26–33). Understanding how pan-sarbecovirus bNAbs interact with Spike could similarly inform epitope-based vaccine strategies, and reliable elicitation of such bNAbs could reduce the need for frequent vaccine updating. Moreover, given the recent history of recurrent zoonotic sarbecovirus spillovers into human populations, having a diverse array of bNAb therapeutics on-hand would contribute to future pandemic preparedness. In addition, there is keen interest in identifying and developing alternative mAb delivery platforms such as mRNA-lipid nanoparticles (LNPs) since clinical utilization of mAbs has been hampered by the high cost of protein production (34).

Here, we report the isolation and characterization of 20 distinct, RBD-directed bNAb lineages from a convalescent, vaccinated participant who was sampled longitudinally. Thirteen of these targeted the highly conserved class 1/4 or class 4 epitopes and used the IGHD3–22 gene segment-encoded YYDxxG motif or a somatic variant thereof (35, 36). The five broadest bNAbs neutralized all 18 viruses in our screening panel, including the recently emerged XBB.1.5 and JN.1 omicron VOCs as well as representatives of other sarbecovirus clades, with a geometric mean titer (GMT) of 0.38 μg/mL IC_50_ (half-maximal inhibitory concentration). Structural analysis of the two most potent class 1/4 bNAbs (Ab401 and Ab568) revealed that they target conserved RBD epitopes, similar to other cross-reactive mAbs isolated from human convalescent donors including COVA1–16, ADI-62113, C022, CC25.54, CC84.24, 10–40, and pT1679 (18–20, 35, 37–41). Prophylactic delivery of Ab401 as either a recombinant protein or mRNA-LNP protected human ACE2-expressing mice from a lethal SARS-CoV-2 challenge as well as that with a related bat sarbecovirus. Together, these data demonstrate that convalescent, vaccinated individuals can generate bNAb responses against viral variants they had never encountered and which had not emerged at the time of sampling. Our results also contribute multiple pan-sarbecovirus bNAbs to the arsenal of therapeutic countermeasures against zoonotic sarbecovirus spillovers and identify mRNA mAb delivery as a promising platform to protect immunocompromised patients against current and future SARS-CoV-2 VOCs.

## Results

### Isolation of pan-sarbecovirus broadly neutralizing antibodies

**Figure 1.**
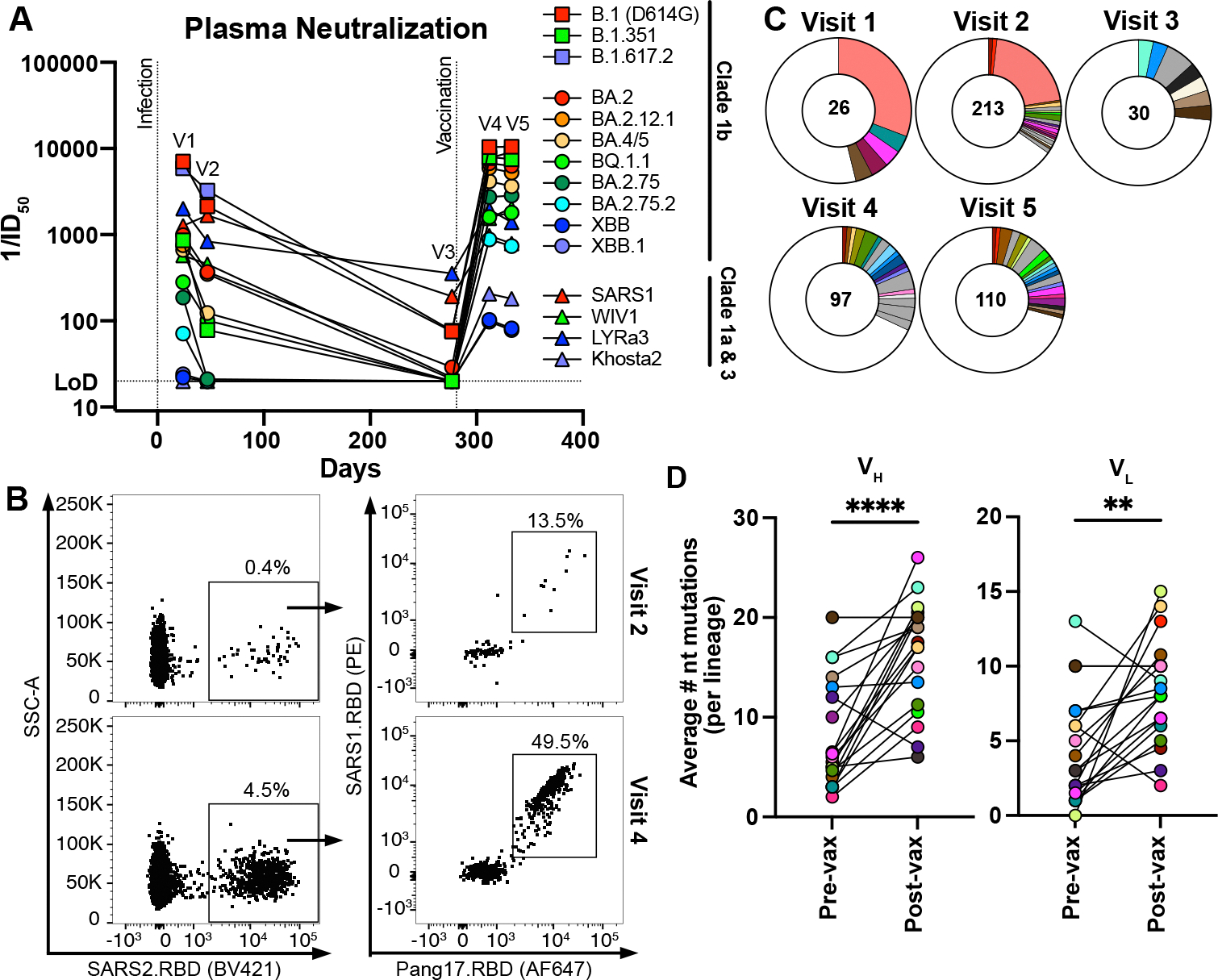
Plasma neutralization and B cell binding breadth increase over time in a convalescent, vaccinated COVID-19 patient. **(A)** Plasma neutralization activity against a multiclade 15-virus panel, expressed as reciprocal ID_50_. Early SARS-CoV-2 VoCs are represented as squares, omicron VoCs as circles, and sarbecoviruses from other clades as triangles. The timepoint of infection and vaccination are indicated with dashed vertical lines and visits are labeled V1 through V5. **(B)** Peripheral blood mononuclear cells (PBMCs) from the indicated timepoint were stained and analyzed by flow cytometry. Left panels are gated on live CD3^−^CD8a^−^CD14^−^CD16^−^CD19^+^IgD^−^IgM^−^IgG/IgA^+^ B cells and show binding to the autologous SARS-CoV-2_WA1 RBD. The fraction of these SARS-CoV-2 RBD^+^ cells capable of binding SARS-CoV and Pang17 RBDs is shown in the right panels. **(C)** B cell receptor clonality plots showing the proportion of sorted cells at each timepoint that belong to an expanded lineage (containing ≥2 members). Lineages that appear at multiple timepoints are color-coded, expanded lineages that only appear at a single timepoint are grey, and singlets are white. The number in the center of each plot represents the total number of antibody heavy/light chain pairs isolated. **(D)** The average number of nucleotide mutations in the V_H_ and V_L_ antibody gene segments is shown for lineages that were found at both pre-vaccination (Visits 1, 2, or 3) and post-vaccination (Visits 4 or 5) timepoints. Lineages are color-coded as in panel **(C)** and Dataset S1. Wilcoxon matched-pairs rank sum test was used to determine statistical significance. ****P < 0.0001; **P < 0.01. Nt, nucleotide.

To identify donors with pan-sarbecovirus bNAbs, we screened plasma from a cohort of convalescing COVID-19 patients recruited from March to May 2020 (42) for neutralizing activity against a panel of 15 diverse sarbecoviruses including recent SARS-CoV-2 VOCs. Plasma from donor CR0011 potently neutralized the SARS-CoV-2 B.1 strain, which was prevalent in the southern United States at the time of infection (43) as well as several VOCs that had not yet emerged at the time of sampling (Fig. 1A, fig. S1A). Titers waned over a nine-month period of convalescence, but both breadth and potency were boosted considerably upon immunization with the Pfizer-BioNTech COVID-19 mRNA vaccine (BNT162b2) in December 2020 (fig. S1B), which encodes prefusion-stabilized SARS-CoV-2 Wuhan-Hu-1 Spike (44–46). Post-vaccine plasma neutralized all 15 viruses in a multiclade panel with a reciprocal geometric mean titer (GMT) ID_50_ of 1724 (range: 98 to 10,393) (Fig. 1A, fig. S1A).

To isolate mAbs capable of recapitulating this broadly neutralizing activity, we sorted antigen-specific B cells from peripheral blood mononuclear cell (PBMC) samples collected pre- and post-vaccination from donor CR0011 and sequenced their antibody genes. We sorted SARS-CoV-2_WA1 RBD single-positive B cells from four timepoints (Visits 1, 2, 3, and 5), yielding 334 paired heavy- and light-chain gene sequences, and SARS-CoV-2_WA1, SARS-CoV, Pang17 RBD triple-positive B cells from two timepoints (Visits 2 and 4), yielding an additional 142 paired sequences (fig. S1C). The frequency of SARS-CoV-2_WA1 RBD-binding peripheral B cells increased from 0.4% post-infection to 4.5% post-vaccination, consistent with an anamnestic recall response (Fig. 1B). Furthermore, the proportion of these SARS-CoV-2 RBD-specific B cells capable of binding heterologous RBDs increased 3.7-fold following vaccination (from 13.5% to 49.5%), indicating that immunization promoted acquisition of breadth in this participant (Fig. 1B). Given that COVID-19 is a mucosal infection, we interrogated both IgA^+^ and IgG^+^ antigen-specific B cell subsets. 44.4% of peripheral class-switched (IgM^−^IgD^−^) SARS-CoV-2 RBD-specific B cells expressed IgA three weeks post-infection (Visit 1), and this fraction declined thereafter and was not boosted by intramuscular immunization (fig. S1D).

The 476 isolated antibody sequences comprised 40 expanded lineages (each containing ≥2 members) and 319 singlets (Fig. 1C, Dataset S1). 19 lineages were found both pre- and post-vaccination, most of which showed an increase in somatic hypermutation over time, particularly in V_H_ (Fig. 1D). This was likely due to continued affinity maturation over the nine-month convalescence period as well as vaccine boosting (15, 47). Indeed, we observed a significant increase in the average number of nucleotide mutations in the V_H_ and V_L_ gene segments of antigen-specific B cells between Visit 2 (six weeks post-infection) and Visit 3 (40 weeks post-infection) (fig. S1E). Although there was no further increase in average number of mutations post-vaccination in bulk antigen-specific B cells (fig. S1E), we did observe accrual of additional mutations in several lineages between Visit 3 (one week before vaccination) and Visits 4/5 (four and seven weeks after vaccination, respectively) (fig. S1F). Most expanded lineages were composed exclusively of IgG, although one IgA-only lineage containing 52 members was identified shortly after infection (Visits 1 and 2). Five other lineages had both IgG and IgA members (Dataset S1).

### Functional characterization of pan-sarbecovirus bNAbs

**Figure 2.**
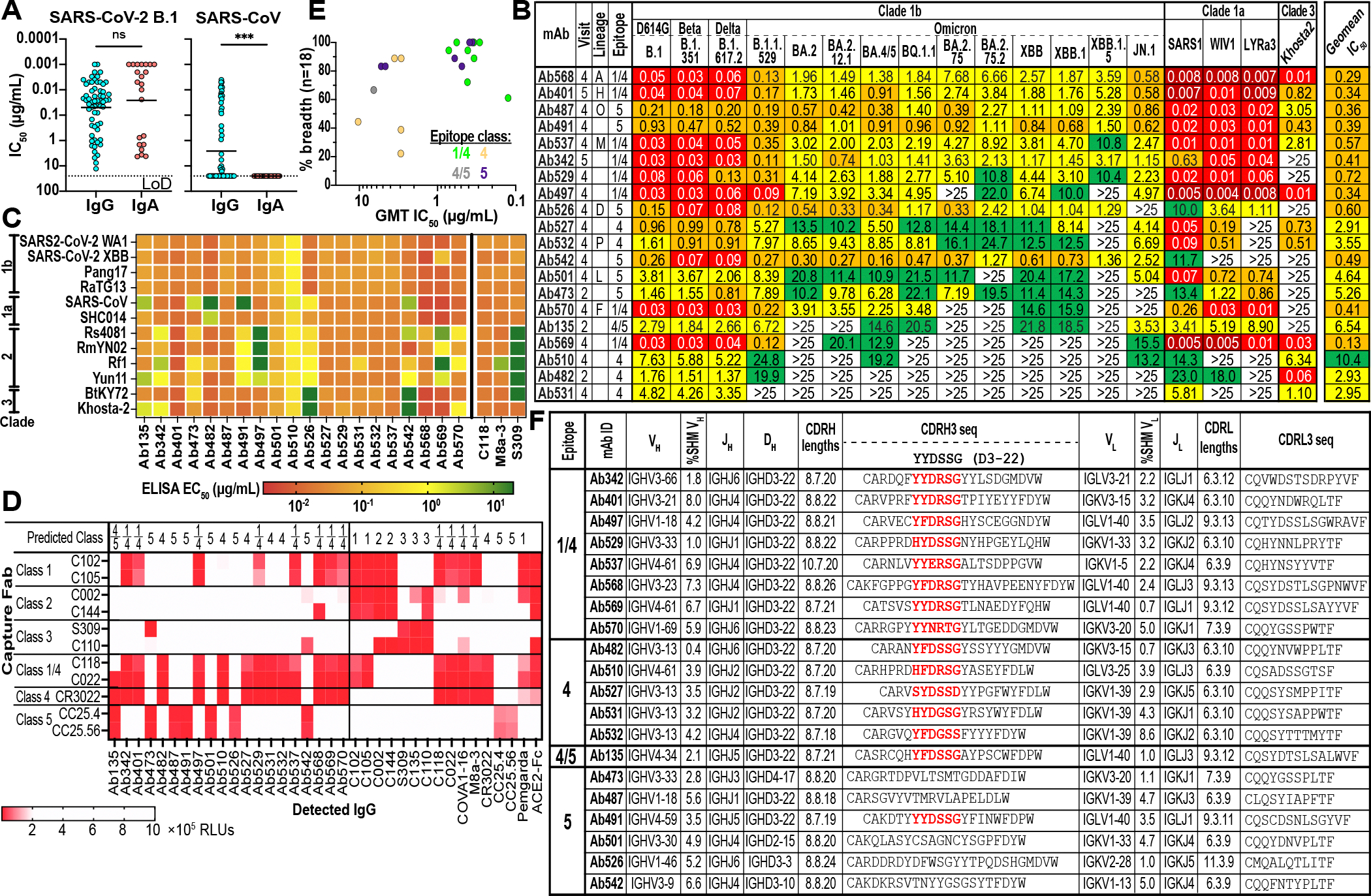
Potent, pan-sarbecovirus broadly neutralizing antibodies target the highly conserved class 1/4 and class 4 RBD epitopes and utilize the IGHD3–22 gene segment. **(A)** Neutralization potency of IgG and IgA monoclonal antibody isolates against SARS-CoV-2_D614G (left) or SARS-CoV (right) pseudovirus, expressed as IC_50_ in μg/mL. Only antibodies that neutralize SARS-CoV-2_D614G with IC_50_ <25 μg/mL are shown. Each circle represents a single antibody and horizontal bars indicate the geometric mean. Mann-Whitney test was used to determine statistical significance. P*** < 0.01; not significant (ns) P > 0.05. **(B)** Neutralization potency of bNAbs representing 20 distinct lineages against a multiclade 18-virus panel, represented as IC_50_ in μg/mL. The visit at which antibody was isolated, lineage designation, and epitope class as determined by competition ELISA (in panel **D**) is provided. **(C)** Heatmap summarizing binding profiles of the 20 bNAbs to diverse sarbecovirus RBDs. Half-maximal effective concentration (EC_50_) in μg/mL for each antibody-RBD pair is shown, as determined by ELISA. Control antibodies include C118 (48), M8a-3 (59), and S309 (21). **(D)** Epitope mapping by competition ELISA. Immobilized Fabs (y-axis) were used to capture SARS-CoV-2_WA1 RBD, and subsequent binding of the indicated IgG antibodies (x-axis) was detected with an anti-Fc secondary. Poor IgG binding (red) indicates competition and may suggest an overlapping epitope with the capture Fab, whereas robust IgG binding (white) indicates lack of competition and a non-overlapping epitope. **(E)** Neutralization breadth and potency of the 20 bNAbs from panel **(B)** against a panel of 18 sarbecovirus strains. Potency is represented as GMT IC_50_ in μg/mL. Each circle represents an individual antibody and the color indicates the corresponding epitope. **(F)** Immunogenetic features of pan-sarbecovirus bNAbs, stratified by epitope class. The IGHD3–22*01 gene segment-encoded YYDSSG motif or somatic variant thereof is highlighted in red font. SHM, somatic hypermutation.

We next synthesized 65 IgG and 25 IgA RBD-binding mAbs and evaluated their neutralization capacity. 62/65 (95%) IgG and 22/25 (88%) IgA mAbs neutralized a pseudovirus bearing the SARS-CoV-2 B.1 Spike (fig. S2), with GMT IC_50_ values of 0.05 μg/mL (IgG) and 0.03 μg/mL (IgA) (Fig. 2A). To down-select antibodies with neutralization breadth, we screened these same mAbs against a pseudovirus bearing the SARS-CoV Spike, which shares only 76% amino acid sequence identity with that of SARS-CoV-2_WA1 (7, 11) and belongs to a different phylogenetic clade (fig. S3). 28 IgG mAbs, but no IgA mAbs, were capable of cross-neutralizing SARS-CoV (Fig. 2A). These differences in cross-neutralization suggested that the IgA mAbs recognize a highly variable region of RBD. To test this hypothesis, we selected four representative IgA-class antibodies (Ab1, Ab19, Ab32, and Ab33) that utilized diverse immunoglobulin genes and potently neutralized SARS-CoV-2_D614G for structural analysis. Negative-stain electron microscopy (nsEM) of Fab-Spike complexes confirmed that these antibodies target the class 1 (Ab19, Ab33) and class 2 (Ab1, Ab32) RBD epitopes (fig. S4).

The 28 cross-neutralizing IgG mAbs represented 20 unique lineages, and a member of each was tested against a multiclade pseudovirus panel expressing 18 diverse sarbecovirus Spikes, including several from SARS-CoV-2 VOC strains that had not emerged at the time this participant was sampled. All 20 mAbs exhibited potent heterologous neutralization activity, and seven neutralized at least 17/18 (94%) viruses in the panel with GMT IC_50_ values ranging from 0.29–0.72 μg/mL (Fig. 2B), on par with the best human pan-sarbecovirus bNAbs (fig. S5) (18, 19, 22, 38, 48). We also evaluated binding to an even more diverse panel of sarbecovirus RBDs, including clade 2 sarbecoviruses that do not use ACE2 as an entry receptor and thus could not be assessed in our neutralization assay. Importantly, our best bNAbs also exhibited broad and potent binding profiles, with all of them recognizing clade 2 RBDs (Fig. 2C), unlike S309 (a derivative of which was licensed as sotrovimab) and Pemgarda (the only currently FDA-approved mAb as of November 2025) (4, 49). Of note, 17 of the 20 bNAbs were isolated using a SARS-CoV-2_WA1, SARS-CoV, Pang17 RBD triple-positive sort strategy, suggesting that heterologous RBD baits enriched for B cell receptor (BCR) binding breadth (Dataset S1). As expected, neither our antibodies nor previously described anti-sarbecovirus RBD antibodies were able to cross-neutralize divergent merbecoviruses such as HKU5 (fig. S5B).

To better understand the basis of the observed binding and neutralization breadth, we mapped the epitopes targeted by our 20 bNAbs via competition ELISA. Competition indicates an inability for two mAbs to bind an antigen simultaneously, which could indicate epitope overlap, steric clashes, or other effects, although in the case of anti-RBD antibodies there has been high concordance between competition- and structure-based epitope classification (49). Fabs from NAbs known to recognize class 1, 2, 3, 1/4, 4, or 5 RBD epitopes (9–14, 18, 20) were coated on a plate and incubated with SARS-CoV-2_WA1 RBD, and the ability of bNAbs of unknown specificities to bind these complexes was queried. A comparison of the binding patterns of our 20 bNAbs to those of well-characterized RBD-specific mAbs revealed five class 4, eight class 1/4, one class 4/5, and six class 5 antibodies (Fig. 2D). This classification was consistent with the observed neutralization phenotype: all mAbs categorized as class 5 had neutralization patterns characteristic of class 5 antibodies, exhibiting moderate neutralization potency with little or no diminution against the omicron VOCs (Fig. 2B) (20, 50, 51). As expected, the class 1/4 and class 5 bNAbs had the greatest breadth and potency (Fig. 2B, E).

**Figure 3.**
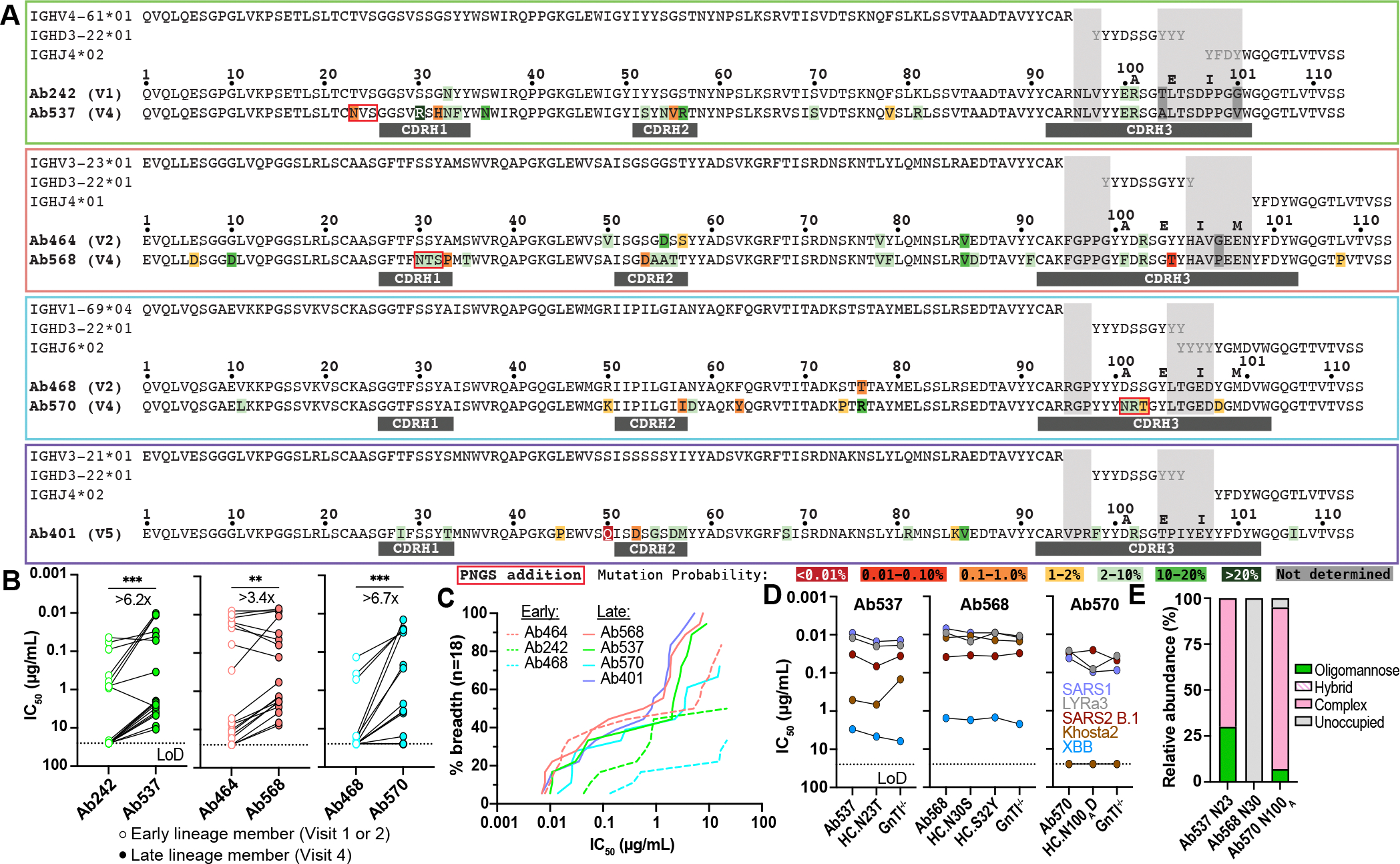
Affinity maturation promotes acquisition of neutralization breadth and potency in pan-sarbecovirus bNAb lineages. **(A)** Amino acid alignments of pan-sarbecovirus bNAb heavy chains with earlier lineage members and the closest germline V_H_, D_H_, and J_H_ alleles from the IMGT database (85, 86). Somatically mutated residues in the bNAb sequences are color-coded by estimated mutation probability, as determined by ARMADiLLO (54). Non-templated regions are highlighted in light grey and were excluded from analysis. Potential N-linked glycosylation site (PNGS) sequons are boxed. Residues are numbered using the Kabat scheme via ANARCI (87). The timepoint of mAb isolation is listed as visit number (V1 through V5). CDR, complementarity determining region. The corresponding light chain alignments are shown in Fig. S7. **(B)** Neutralization potency of class 1/4 antibody pairs against an 18-virus panel, comparing early and late members of the same three lineages. Each circle represents neutralization of an individual pseudovirus with open circles representing the early lineage member and closed circles representing the late lineage member, represented as IC_50_ in μg/mL. Antibodies that do not neutralize at ≤ 25 ug/mL are conservatively considered to have IC_50_ = 25 μg/mL for this analysis. The median fold-change in IC_50_ for each antibody pair is listed at the top of the graph. Wilcoxon matched-pairs rank sum test was used to determine statistical significance. ***P < 0.001; **P < 0.01. **(C)** Neutralization breadth and potency curves for the seven bNAbs from panel **(A)** against an 18-virus panel, representing the fraction of viruses neutralized (y-axis) at any given IC_50_ (x-axis). Dotted lines indicate early lineage members and solid lines indicate late lineage members. **(D)** Neutralization activity of the indicated antibody grown in wildtype or *GnTI*^−/−^ 293F cells, or the indicated PNGS knockout mutant antibody. Each circle represents neutralization activity of the mAb against the indicated pseudovirus, expressed as IC_50_ in μg/mL. **(E)** Grouped site-specific glycan analysis of somatically mutated mAbs. Glycan compositions at the indicated heavy chain residue of each mAb are grouped into their corresponding categories, with complex-type glycans displayed in pink, hybrid in hatched pink, oligomannose in green, and unoccupied in grey.

The 20 pan-sarbecovirus bNAbs had relatively long CDRH3 loops (18–26 amino acids) and were immunogenetically diverse, using a wide range of V_H_ and V_L_ gene segments (Fig. 2F). However, all class 1/4, 4, and 4/5 bNAbs utilized the IGHD3–22 gene segment in reading frame 2. This gene segment encodes a YYDxxG motif that facilitates RBD recognition by extending an RBD β-sheet via main-chain hydrogen bonding (18, 20, 35, 36, 39, 40, 49). Our best class 1/4 bNAbs (Ab401, Ab568, and Ab537), which potently neutralized 18 of 18 (100%) viruses in our panel, all acquired an S-to-R substitution within this motif, which enables sidechain hydrogen bonding to RBD in several class 1/4 bNAbs (18, 35, 39). Other substitutions in this motif were generally conservative, including Y100_A_F and D100E in Ab568 and Ab537, respectively (Fig. 2F, Fig. 3A).

### Maturation of class 1/4 bNAb lineages

To better characterize lineage evolution and breadth acquisition, we compared the neutralization profiles and sequence features of early (pre-vaccine) versus late (post-vaccine) bNAb lineage members. We identified early members of three class 1/4 bNAb lineages: Ab242, Ab464, and Ab468 (isolated from Visits 1 and 2) were clonal relatives of Ab537, Ab568, and Ab570 (isolated from Visit 4), respectively (Fig. 3A). All three lineages exhibited an increase in somatic hypermutation over time (Fig. 3A, fig. S1F), indicating ongoing affinity maturation. Consistent with these findings, neutralization breadth and potency increased as well, with late lineage members exhibiting up to a 1094-fold enhancement in IC_50_ titer, with median fold-change ranging from 3.4- to 6.7-fold across lineages (Fig. 3B, C). The median fraction of viruses neutralized by early versus late lineage members rose from 9/18 (50%) to 18/18 (100%) (Fig. 3C), and this increase was primarily due to acquisition of neutralization activity against omicron VOCs (fig. S6).

The maturation of bNAb lineages recognizing other viruses, such as HIV-1, often depends on acquisition of rare or improbable mutations to achieve neutralization breadth (30, 52, 53). To determine whether this trend applied to our pan-sarbecovirus bNAbs, we used the ARMADiLLO computational pipeline to evaluate the probability of each somatic mutation (54). ARMADiLLO accounts for both the number of nucleotide substitutions required to make a nonsynonymous change as well as the preferential targeting of certain sequence motifs by activation-induced cytidine deaminase (AID) (54). In addition to Ab537, Ab568, and Ab570, we also included Ab401 in this analysis as it was one of the broadest class 1/4 bNAbs. Interestingly, although these four mAbs were isolated following prolonged antigen exposure (i.e. more than ten months after SARS-CoV-2 infection with an intervening vaccination), 56/91 (62%) of amino acid substitutions in the heavy and light chain templated regions were predicted to be probable (Fig. 3A, fig. S7). Notably, the S-to-R change in the IGHD3–22-encoded YYDxxG motif was predicted to be probable in all four lineages (Fig. 3A). Together, these results suggest that once primed, such class 1/4 bNAb lineages have relatively simple maturational trajectories to breadth and potency, making them ideal vaccine targets.

Over the course of affinity maturation, three of the class 1/4 bNAb lineages acquired potential N-linked glycosylation sites (PNGSs) in or adjacent to CDRH1 (Ab537, Ab568) or CDRH3 (Ab570) (Fig. 3A). Somatic hypermutation introduced all three amino acid residues of the NxT/NxS PNGS sequon in Ab568 and Ab570, but only the N residue in Ab537 (Fig. 3A). To determine whether glycans at these sites impact RBD recognition, we reverted the substituted N, S, or T residues back to the germline-encoded residue and assessed neutralization against a five-member sarbecovirus panel. In parallel, we produced Ab537, Ab568, and Ab570 in *GnTI*^−/−^ cells, which lack N-acetylglucosaminyltransferase I and thus have a global deficiency in glycan processing (55, 56). Neutralization activity was not affected by any of these modifications (Fig. 3D), suggesting either that the PNGS sequons introduced by somatic hypermutation are not glycosylated or that glycans at these sites do not contribute to RBD recognition.

To assess N-glycosylation at these somatic hypermutation-introduced PNGSs, we performed site-specific glycan analysis by mass spectroscopy. The Ab568 N30 PNGS was almost completely glycan-devoid (Fig. 3E), consistent with its proximity to the proline residue at position 33 (Fig. 3A), as prolines immediately adjacent to PNGS sequons adversely affect N-glycan occupancy (26, 57). In contrast, the Ab537 N23 and Ab570 N100_A_ PNGSs were almost fully occupied. These glycans were primarily complex-type bi- and tri-antennary glycans, although a small fraction were oligomannose-type (Fig. 3E, fig. S8A). This under-processed oligomannose glycan fraction was largely composed of Man_5_GlcNAc_2_ (M5) in both antibodies, suggesting that while there may be an element of local steric restraint imparted by the surrounding protein architecture, these glycans are largely accessible to the glycan-processing machinery, with only a residual population escaping full maturation. The detected complex-type glycans were highly fucosylated in both Ab537 and Ab570 (fig. S8B). While our mass spectrometry method does not reveal precise glycan structure or isometry, glycans containing a single fucose moiety are likely core-fucosylated, while those containing >1 moiety are likely outer arm-fucosylated (Dataset S2). Interestingly, sialic acid could only be detected on Ab570, in which ~60% of glycans at position N100_A_ were modified by at least one NeuAc monosaccharide (fig. S8B). Similarly, sulfated glycans could only be detected on Ab570, albeit at low abundance (fig. S8B). Taken together, the presence of these glycan modifications along with the low proportion of oligomannose-type glycans in Ab570 suggest that the position of this acquired PNGS allows glycans to be more heavily modified and elongated compared to that of Ab537.

### Structural characterization of class 1/4 bNAbs

**Figure 4.**
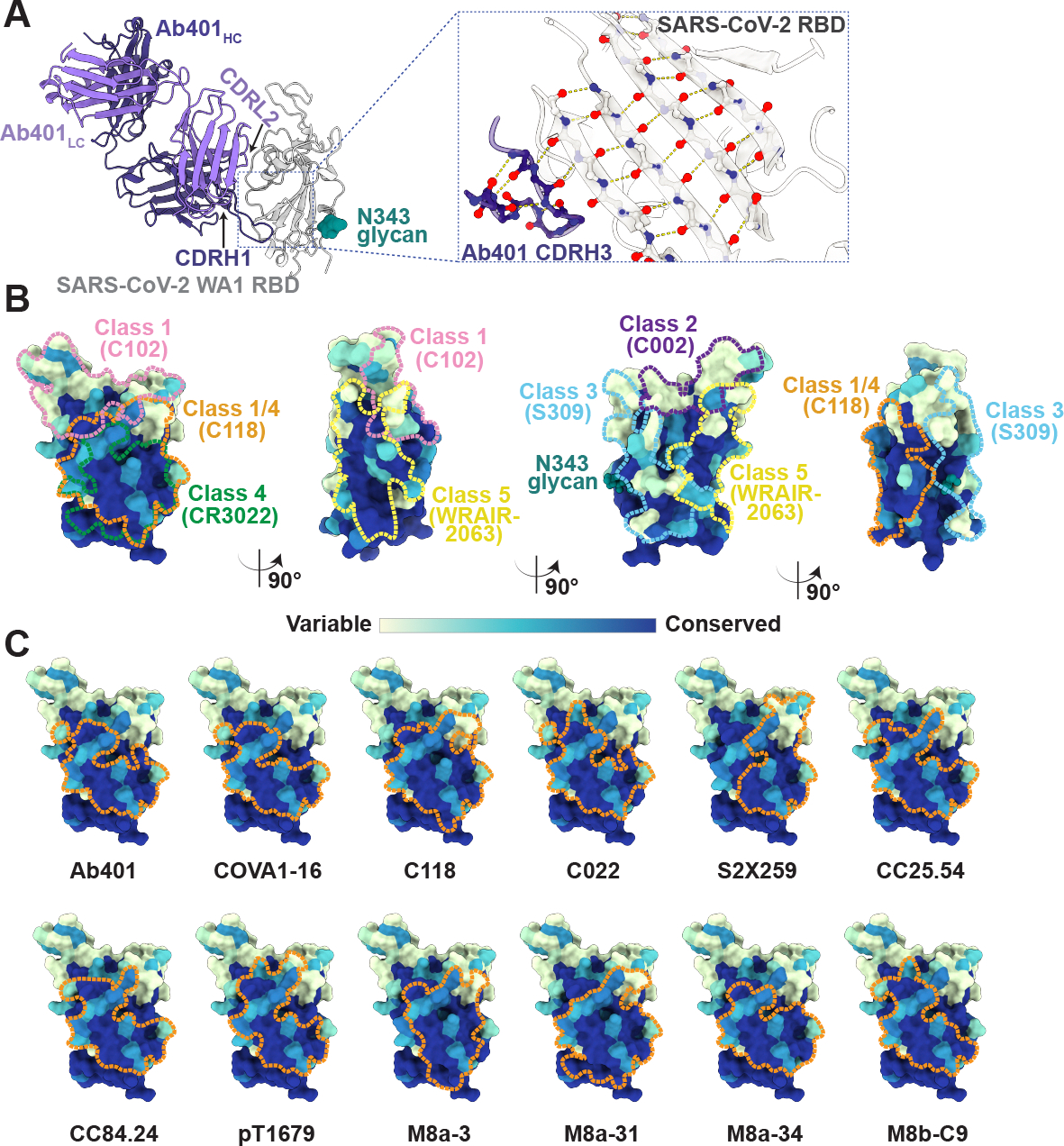
Structural analysis of class 1/4 pan-sarbecovirus bNAbs reveals similar conserved RBD epitopes. **(A)** Crystal structure of the Ab401 Fab–SARS-CoV-2 RBD complex. Hydrogen bonds between the CDRH3 and RBD are shown as dotted yellow lines in the insert. **(B)** Sequence conservation of 16 sarbecovirus RBDs (SARS-CoV-2, RShSTT200, Pang17, RaTG13, SARS-CoV, WIV1, SHC014, LYRa3, C028, Rs4081, RmYN02, RF1, Yun11, BM4831, BtKY72, and Khosta2) calculated using the Consurf Database (88) plotted on a SARS-CoV-2 RBD surface diagram (PDB 7BZ5). RBD epitopes as determined by PDBePISA (89) are outlined in different colors based on structures of representatives Ab-RBD complexes (C102: PDB 7K8M, C002: PDB 7K8T, S309: PDB 7JX3, CR3022: PDB 7LOP, C118: PDB 7RKV; and WRAIR-2063: PDB 8EOO). **(C)** Comparison of class 1/4 epitopes of human, mouse, and rabbit mAbs. Ab401: 9ZDU (this study); COVA1–16: PDB 7S5R; C118: PDB 7RKV; C022: PDB 7RKU; S2X259: PDB 7M7W; CC25.54: PDB 8SIR; CC84.24: PDB 8SIT; pT1679: PDB 9H6U; M8a-3: PDB 7UZ4; M8a-31: PDB 7UZ7; M8a-34: PDB 7UZC; and M8b-C9: PDB 9ML9.

To determine the structural basis of pan-sarbecovirus recognition by one of our best class 1/4 bNAbs, we solved a 2.6 Å resolution crystal structure of Ab401 bound to SARS-CoV-2_WA1 RBD (Fig. 4A, Table S1) (58). The Ab401-RBD crystal structure revealed that Ab401 recognizes the class 1/4 sarbecovirus RBD epitope (Fig. 4B), consistent with the competition ELISA data indicating that Ab401 competes with both class 1 and 4 anti-RBD mAbs for binding to SARS-CoV-2 RBD (Fig. 2D). Ab401 interacts with the RBD through its CDRH1, CDRH3, and CDRL2 loops with most interactions contributed by the CDRH3 loop that extends a β-sheet network in the RBD (Fig. 4A). In a binding footprint analysis, Ab401 targets a conserved region on the RBD (Fig. 4C, fig. S9) that is similar to the binding epitopes of class 1/4 human anti-RBD mAbs elicited by infection, including COVA1–16, C118, C022, S2X259, CC25.54, CC84.24, and pT1679 (18–20, 35, 37–40), and mAbs elicited in experimental animals by immunization, including M8a-3, M8a-31, M8a-34, and M8b-C9 (Fig. 4C) (49, 59). Recognition of the conserved class 1/4 epitope is consistent with the broad and potent neutralization (Fig. 2B) and binding (Fig. 2C) profiles of Ab401.

We also characterized Ab568 in complex with SARS-CoV-2_WA1 Spike using single-particle cryoEM (60). Although an atomic model of the complex could not be built due to low resolution of the EM density (5.2 Å) (Table S2), we predicted the RBD epitope of this mAb by docking an AlphaFold 3 (61) model of Fab and Spike RBD into the density, which showed three Fabs interacting with RBDs in “up” conformations (fig. S10). This modeling predicted that much like Ab401, Ab568 also recognizes the class 1/4 RBD region, consistent with its broad binding (Fig. 2C), potent neutralization (Fig. 2B), and competition (Fig. 2D) profiles.

**Figure 5.**
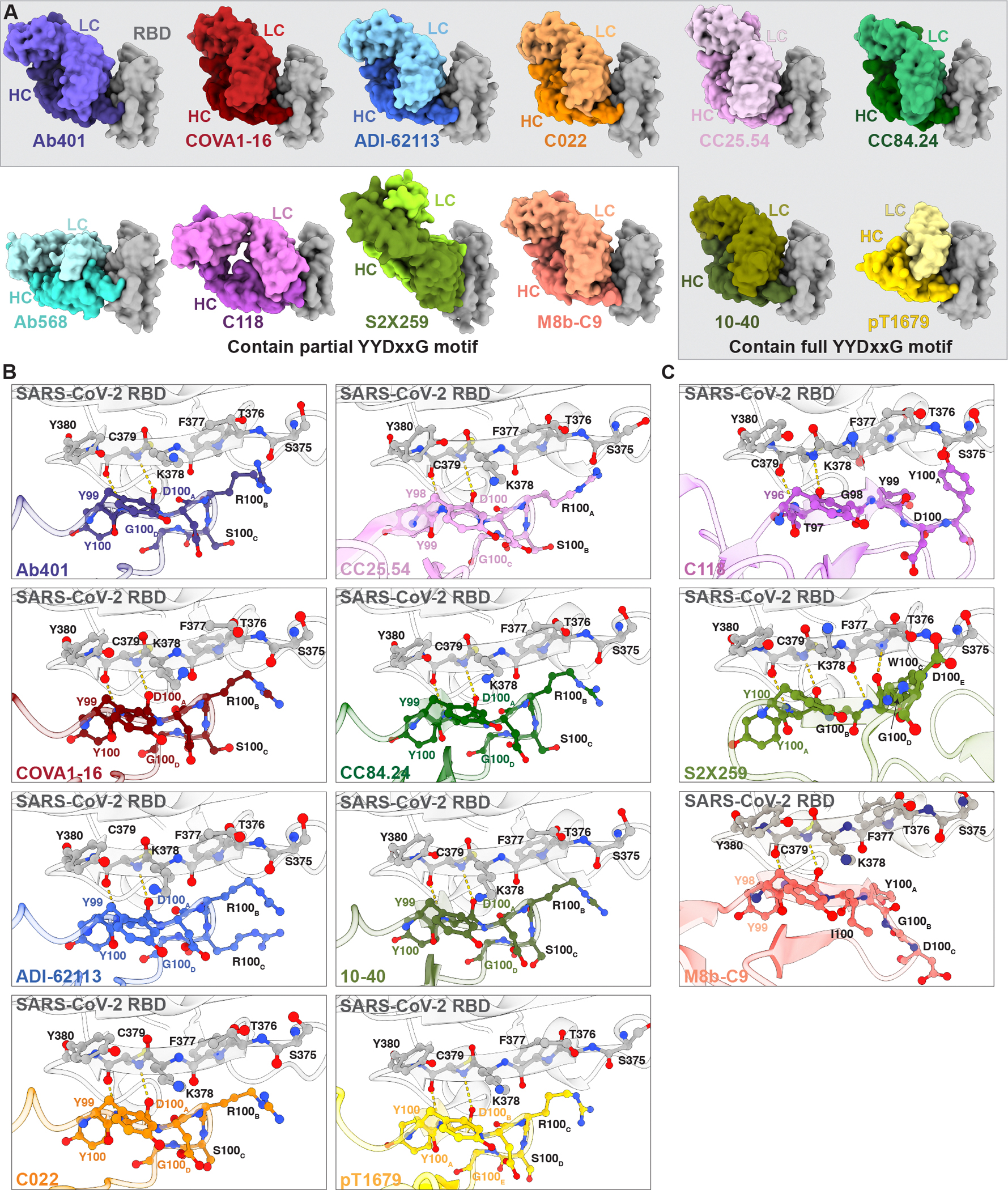
Comparison of class 1/4 anti-RBD mAbs containing YYDxxG, YY, and Y sequence motifs in CDRH3. (**A**) Angle of approach comparison of Fabs from class 1/4 mAbs (various colors) bound to the SARS-CoV-2 RBD (gray). Ab401 and Ab568 (from this study) are shown to the left. **(B, C)** Comparison of backbone hydrogen bonds (yellow dotted lines) between SARS-CoV-2 RBD and mAbs that include a YYDxxG sequence (**B**) or a YY or Y sequence (**C**) in their CDRH3s. Ab401: 9ZDU (this study); COVA1–16: PDB 7S5R; ADI-62113: PDB 7T7B; C022: PDB 7RKU; CC25.54: PDB 8SIR; CC84.24: PDB 8SIT; Ab568: AlphaFold 3 model (this study); 10–40: PDB 7SD5; pT1679: PDB 9H6U; S2X259: PDB 7M7W; C118: PDB 7RKV; and M8b-C9: PDB 9ML9.

We also used the Ab401-RBD crystal structure to compare RBD recognition properties with those of other class 1/4 mAbs isolated from convalescent donors (Fig. 5). Among these human mAbs, Ab401 shares the same angle of approach as mAbs COVA1–16, ADI-62113, C022, CC25.54, CC84.24, 10–40, and pT1679 (Fig. 5A), all of which include a YYDxxG motif in their CDRH3 loops (Fig. 5B). In the Ab-RBD structures of Ab401 and other YYDxxG-containing mAbs (Fig. 5B), the YY residues (Y99 and Y100 in Ab401, COVA1–16, ADI-62113, C022, 10–40, CC84.24; Y98 and Y99 in CC25.54; and Y100 and Y100_A_ in pT1679) form backbone hydrogen bonding interactions with RBD residues 378–379 of a mainchain β-strand to extend an RBD β-sheet (Fig. 4A, 5B). Antibodies that include only a part of the motif (Y100 and Y100_A_ in S2X259; Y96 and T97 in C118) also extend the RBD β-sheet by making mainchain hydrogen bonding interactions with the backbone of RBD residues 378–379 (Fig. 5C), though their angles of approach differ from YYDxxG motif-containing mAbs (Fig. 5A). The AlphaFold-predicted structure of the Ab568-RBD complex suggested that Ab568 has a similar angle of approach as C118 (Fig. 5A). In addition, M8b-C9, a mAb isolated from an immunized rabbit (49), also includes a YY motif in its CDRH3, with Y98 and Y99 forming backbone hydrogen bonds with RBD residues 378–379 (Fig. 5C) and exhibiting a similar angle of approach as YYDxxG motif-containing mAbs like Ab401 (Fig. 5A). Interestingly, the YY sequence in M8b-C9 is derived from N-region addition rather than being D gene segment-encoded (49).

### Modelling of N-glycans acquired by somatic hypermutation in bNAbs

**Figure 6.**
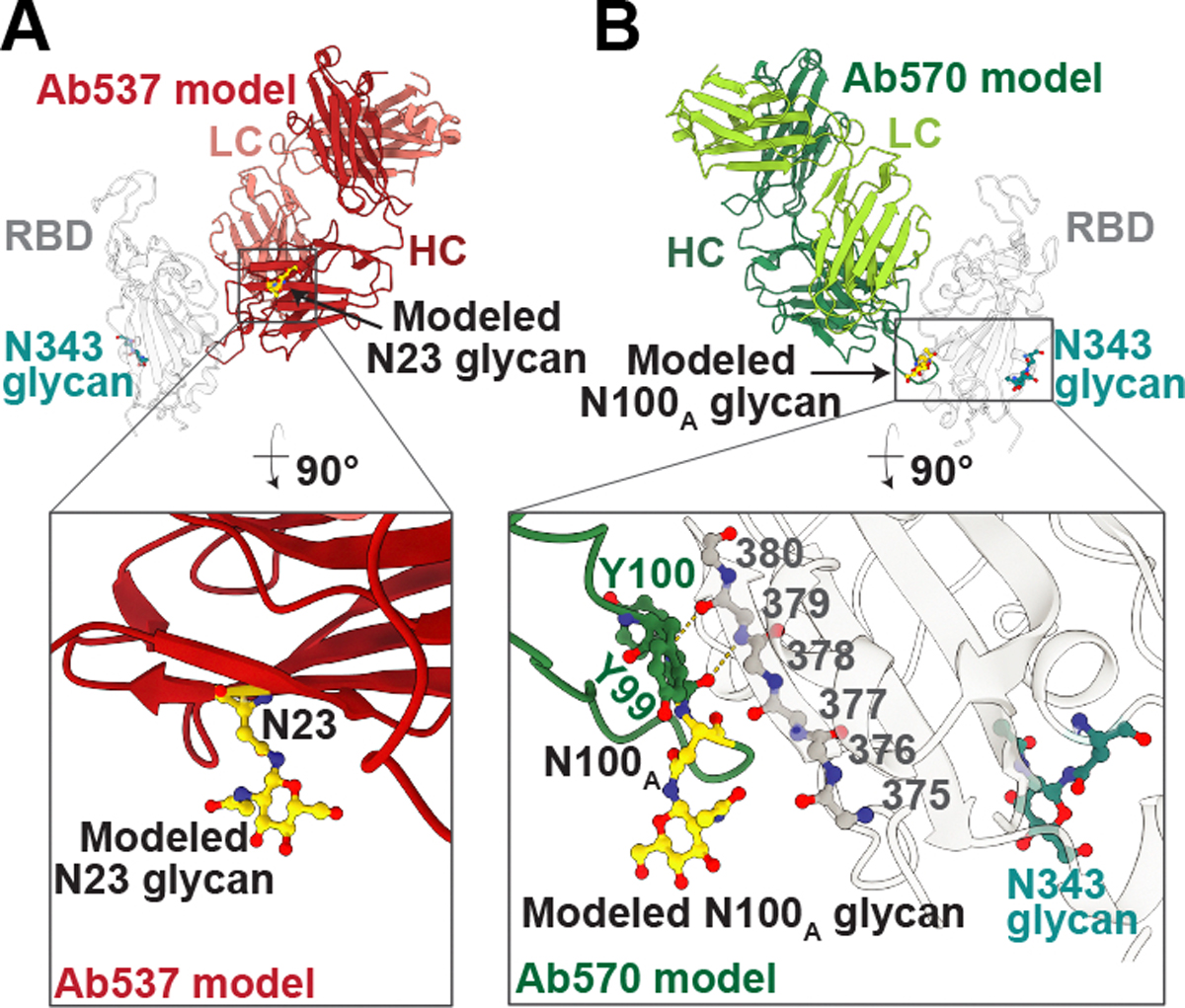
N-linked glycans in Ab537 and Ab570 structural models do not appear to affect RBD recognition. **(A, B)** Modeling the effects of N-glycans in Ab537 (**A**) and Ab570 (**B**) on RBD recognition. Coordinates of the Ab401–SARS-CoV-2 RBD complex structure were used to model an Asn residue and an attached N-acetylglucosamine at the PNGSs in Ab537 (**A**) and Ab570 (**B**). Atom coloring: Red, oxygen; blue, nitrogen; gray (RBD), yellow (glycan) or teal (glycan), carbon. Mainchain hydrogen bonds are shown as yellow dots in (**B)**. Top: N-linked glycan modeling of Ab537–RBD (**A**) and Ab570–RBD (**B**) based on the Ab401-RBD complex at heavy chain position 23 (**A**) and 100_A_ (**B**). Bottom: Zoomed-in views of modeled N-glycans. (**A**) An N-glycan at position 23 before the CDRH1 of Ab537 is predicted to be solvent-exposed and therefore not at the interface with an RBD. (**B**) An N-glycan at position 100_A_ after the YY sequence in the CDRH3 of Ab570 is not predicted to contact RBD or interfere with binding, suggesting that the presence of an N-glycan in the CDRH3 of a class 1/4 anti-RBD Ab with a YY sequence would not affect RBD recognition.

To understand the molecular basis of how N-glycans in class 1/4 anti-RBD antibodies could affect RBD recognition, we used the crystal structure of the Ab401–SARS-CoV-2 RBD complex (Fig. 4A) to model the locations of N-glycans in representative N-glycosylated antibodies characterized by mass spectroscopy (Fig. 3E): Ab537, which includes an N-glycan attached to heavy chain residue N23 located N-terminal to its CDRH1 loop (Fig. 6A), and Ab570, which includes an N-glycan attached to heavy chain residue N100_A_, which is immediately C-terminal to the YY sequence in its CDRH3 loop (Fig. 6B).

The Ab537 model predicts that the N23 N-glycan (modeled as N-acetylglucosamine) is solvent-exposed and not at the RBD interface (Fig. 6A), consistent with the comparable neutralization potencies observed for Ab537 and its N23T PNGS knockout mutant (Fig. 3D). We also modeled an N-linked glycan at position N100_A_ in the Ab570 CDRH3 loop, immediately downstream of the YY sequence (Fig. 6B). This modeled glycan was also predicted to be solvent-accessible and to not interfere with the hydrogen bonding interactions made between the mainchain atoms of the CDRH3 YY residues and the backbone of the RBD β-strand (Fig. 6B), an interaction that is conserved in class 1/4 anti-RBD antibodies with YYDxxG and YY motifs (Fig. 5B–C) (49). The model demonstrates that an N-linked glycan at residue N100_A_ in the Ab570 CDRH3 would not block recognition of RBD, consistent with the lack of change in neutralization potency between Ab570 and its N100_A_D PNGS knockout mutant (Fig. 3D). These results demonstrate that class 1/4 bNAbs containing YYDxxG or YY motifs that form mainchain hydrogen bonds with RBD, rather than those using sidechains at the tip of their CDRH3, can include PNGSs without disrupting CDRH3 interactions with RBD.

### Prophylactic administration of Ab401 as protein or mRNA protects against challenge with diverse sarbecoviruses

Given the utility of passive immunization in clinical management of immunocompromised individuals, we next assessed whether prophylactic administration of Ab401 could prevent infection in a challenge model. Developing and manufacturing recombinant mAbs for therapeutic applications is costly and can limit clinical use in resource-poor settings, and as such we explored an alternative, mRNA-based delivery system. Nucleoside-modified mRNA constructs encoding the heavy and light chains of Ab401 were generated and co-encapsulated in lipid nanoparticles (LNPs).

**Figure 7.**
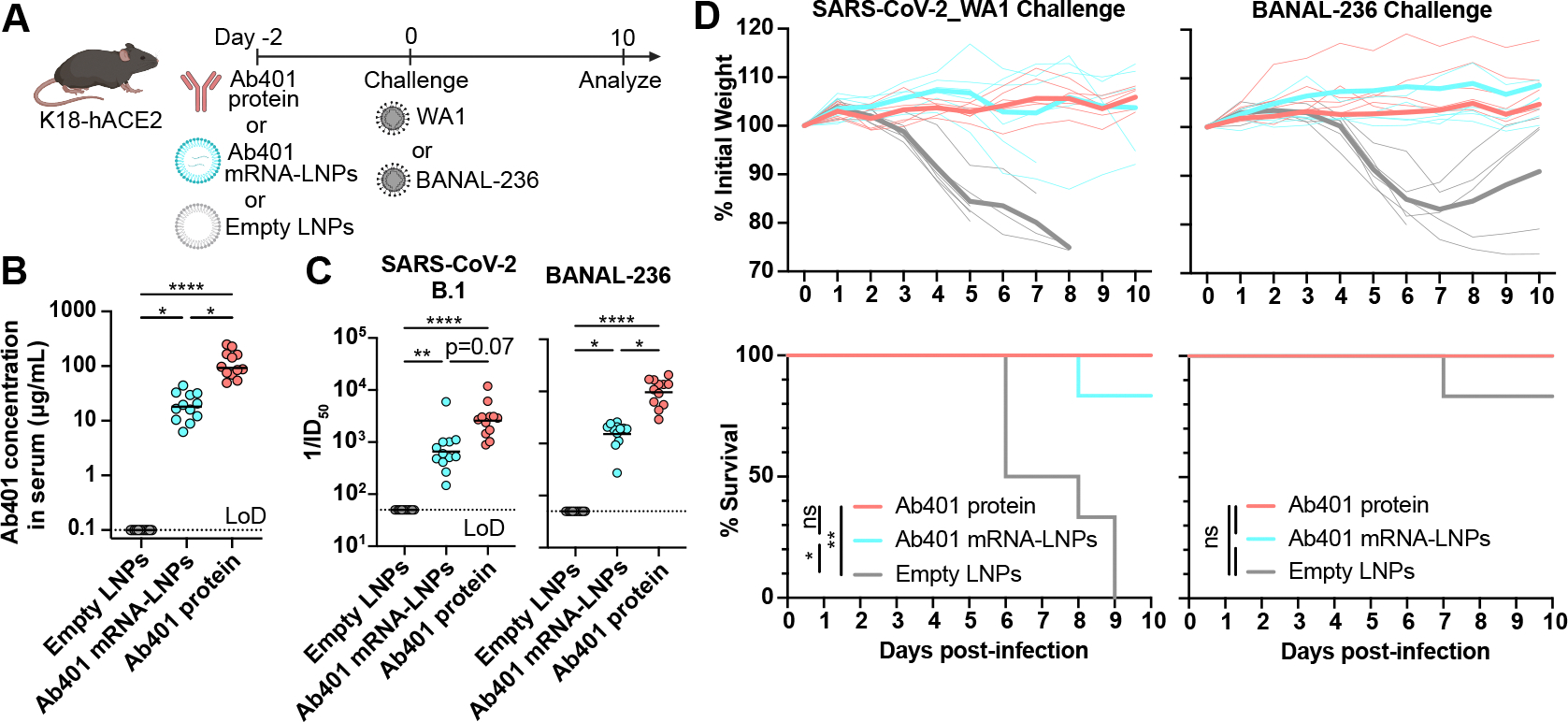
Prophylactic administration of Ab401 as a recombinant protein or mRNA-LNP protects against challenge with diverse sarbecoviruses in a mouse model. **(A)** Schematic illustrating the experimental setup. Recombinant Ab401 (20 mg/kg), LNP-encapsulated mRNA encoding Ab401 heavy and light chains (30 μg per mouse), or empty LNPs (at an equivalent lipid concentration to the mRNA-LNPs) were administered retro-orbitally to K18-hACE2 mice, which express human ACE2 in epithelial cells under the keratin18 (K18) promoter. Two days later, the mice were challenged intranasally with 10^6^ plaque-forming units (PFU) of SARS-CoV-2_WA1 or BANAL-236 virus and were followed for ten days. All intervention/virus pairs had n=6 mice. **(B)** Concentration of functional Ab401 in the serum of passively immunized mice was determined by ELISA, presented as μg Ab401 per mL plasma. **(C)** Neutralization activity of serum collected at day 0 (immediately prior to challenge) against SARS-CoV-2_D614G and BANAL-236 pseudoviruses, represented as reciprocal ID_50_. Each circle represents an individual mouse and horizontal bars represent the geometric mean. Kruskal-Wallis test with Dunn’s test for multiple comparisons was used to determine statistical significance. P**** < 0.0001; **P < 0.01; *P < 0.1. **(D)** Weight loss curves (top) and survival curves (bottom) of the mice from panel **(A)**. Mantel-Cox log-rank test with Bonferroni correction for multiple comparisons was used to determine statistical significance of the survival data. **P < 0.01; *P < 0.1; not significant (ns) P > 0.05.

To test whether passive immunization with Ab401 affords protection *in vivo*, we retro-orbitally administered recombinant Ab401 mAb (20 mg/kg), Ab401-encoding mRNA-LNPs (30 μg per mouse), or empty LNPs (at an equivalent lipid concentration to the mRNA-LNPs) to transgenic K18-hACE2 mice, which express human ACE2 in epithelial cells (62, 63) (Fig. 7A). Two days later, mice were intranasally challenged with 10^6^ plaque forming units (PFU) of either SARS-CoV-2_WA1 or the related bat sarbecovirus BANAL-236 (fig. S3), which has zoonotic potential (64). The median serum concentration of Ab401 immediately prior to challenge was 92 μg/mL in the protein recipient group and 18 μg/mL in the mRNA-LNP recipient group (Fig. 7B), more than two orders of magnitude above the Ab401 IC_50_ of 0.043 μg/mL against SARS-CoV-2_D614G (Fig. 2B). As expected, serum from this same timepoint from both groups of Ab401 recipient mice potently neutralized the challenge viruses, whereas that from control mice did not (Fig. 7C). Consistent with their higher serum concentration, mice that received Ab401 as a recombinant mAb had reciprocal GMT ID_50_ titers of 2,578 and 9,666 for SARS-CoV-2_D614G and BANAL-236, respectively (Fig. 7C), while those that received Ab401 as mRNA-LNPs had 3.9-fold lower plasma neutralizing titers against SARS-CoV-2_D614G (p=0.06) and 6.3-fold lower titers against BANAL-236 (p=0.01) (Fig. 7C). Upon challenge with SARS-CoV-2_WA1, all control mice rapidly lost body weight, succumbed to infection, and died within nine days (Fig. 7D). In contrast, mice that had received prophylactic Ab401 maintained their pre-challenge body weight and exhibited significantly enhanced survival, with 6/6 mice in the recombinant protein group and 5/6 mice in the mRNA-LNP group surviving until day ten (Fig. 7D). Ab401 administration also protected against challenge with the less-pathogenic BANAL-236 virus, with all mice maintaining their body weight and surviving regardless of Ab401 delivery platform (Fig. 7D). In contrast, control mice exhibited substantial weight loss upon BANAL-236 challenge, with one dying at day seven (Fig. 7D). Together, these results demonstrate that a single dose of a potent class 1/4 bNAb can prevent infection with diverse sarbecoviruses and establish mRNA-LNPs as an effective delivery system for mAb therapeutics.

## Discussion

Despite the initial promise of mAb therapies in treating and preventing SARS-CoV-2 infection, most have become obsolete due to viral evolution and escape. The isolation and characterization of bNAbs that recognize conserved epitopes will be critical to developing more permanent pan-sarbecovirus bNAb therapeutics, which could provide immediate relief to immunocompromised individuals whose current clinical options are limited. Furthermore, understanding routes of bNAb maturation and modes of RBD recognition will be key to designing vaccine schema that protect against future VOCs and spillover events.

Here, we studied the development of 20 bNAb lineages in a participant with hybrid immunity. Representatives of all 20 lineages targeted the highly conserved class 1/4, 4, 5, or 4/5 RBD epitopes and potently neutralized diverse SARS-CoV-2 VOCs as well as sarbecoviruses from other clades. Interestingly, all antibodies that competed with canonical class 4 antibody CR3022 (comprising our class 4, 1/4, and 4/5 bNAbs) shared an IGHD3–22-encoded YYDxxG motif in their CDRH3 loops. The five broadest bNAbs neutralized 18 of 18 (100%) diverse viruses with GMT IC_50_ values ranging from 0.3–0.6 μg/mL. Structural characterization of the two most potent, Ab401 and Ab568, confirmed that they target the class 1/4 RBD epitope with a similar angle of approach as other class 1/4 bNAbs. Moreover, Ab401 made similar epitope-paratope interactions as other YYDxxG motif-containing class 1/4 bNAbs, using its CDRH3 to form mainchain hydrogen bonds with RBD residues 378–379 and extend an RBD β-sheet (18–20, 35, 37–40). Prophylactic administration of Ab401 as either recombinant protein or mRNA-LNPs afforded robust protection against challenge with SARS-CoV-2 or BANAL-236, a bat sarbecovirus with zoonotic potential.

Our data indicate that class 1/4 bNAbs represent promising targets for vaccine elicitation. Given that passive immunization with Ab401 protected against infection with diverse sarbecoviruses (Fig. 7D), reliable induction of a polyclonal class 1/4-targeted response by vaccination could mitigate future zoonotic spillover events and reduce the need for frequent vaccine updating in the context of the COVID-19 pandemic. Our data confirm and extend findings from others that infection and/or vaccination with the ancestral SARS-CoV-2_WA1 can induce class 1/4 antibodies (19, 22, 38–40, 48), although these responses are subdominant compared to class 1 and 2 responses targeting poorly conserved RBD regions (65), particularly in the absence of subsequent boosting. Once primed, however, class 1/4 antibodies may have relatively straightforward maturation pathways to breadth, as evidenced by their relatively low rates of somatic hypermutation (0.4–8.0% in V_H_ at the nucleotide level) (Fig. 2F). Furthermore, the majority of amino acid substitutions in our best class 1/4 bNAbs were predicted to be “probable” (Fig. 3A), in contrast to most anti-HIV bNAbs, which tend to have complex developmental trajectories characterized by high rates of somatic mutation and improbable mutations (30, 52, 53). Even early class 1/4 lineage members with minimal somatic mutation, such as Ab242, Ab464, and Ab468, exhibited some neutralization breadth (fig. S6), suggesting that immunization with diverse sarbecovirus Spikes may preferentially expand such lineages and immunofocus the response to this conserved epitope (20).

Immunogenetically, class 1/4 antibodies are an attractive vaccine target because they represent a public antibody class that utilizes the IGHD3–22 gene segment (Fig. 2F) and as such might be amenable to induction by germline-targeting immunogen design strategies (24, 36), which have shown success at inducing V_H_1–2 gene segment-utilizing, VRC01-like anti-HIV bNAbs (32, 66, 67). The bNAbs described here can be used in conjunction with previously characterized bNAbs to iteratively design immunogens that preferentially engage the D gene segment-encoded YYDSSG motif of class 1/4 bNAb precursors while accommodating diverse usage of V and J gene segments. A potential risk of this approach is that individuals lacking the permissive allele may be nonresponders, as was the case in a recent clinical trial aimed at eliciting VRC01-like anti-HIV antibodies (66). Nevertheless, structurally analogous class 1/4 antibodies have been elicited in mice and rabbits, which lack IGHD3–22 homologues, via immunization with mosaic nanoparticles displaying eight heterologous sarbecovirus RBDs (49, 59, 68). Thus, the mosaic nanoparticle platform has the potential to induce class 1/4 bNAbs in diverse populations with heterogenous immunoglobulin repertoires.

The bNAbs described here further expand the armamentarium of antibodies available for clinical development. The withdrawal of almost all previously licensed anti-SARS2 mAbs has left immunocompromised individuals with few clinical options. Indeed, as of November 2025, Pemgarda is the only mAb licensed for clinical use in the USA (6), and recent VOCs including KP.3.1.1 are already exhibiting partial resistance (69, 70). As such, there is an urgent need to identify and develop new mAb therapeutics. Our results identify five bNAbs (Ab568, Ab401, Ab487, Ab491, and Ab537) with pan-sarbecovirus broadly neutralizing activity, all of which could be candidates for clinical development. Passive immunization trials in people living with HIV have suggested that combination therapy with multiple bNAbs targeting different epitopes are superior to monotherapy and may limit viral escape (71–73). To date, most licensed anti-SARS2 mAb therapeutics have been either monotherapy or dual therapy in which one or both mAbs target the variable class 1 or 2 epitopes (4). Going forward, it may be advantageous to develop cocktails of pan-sarbecovirus bNAbs targeting various conserved epitopes (for example, the class 1/4 and 5 RBD epitopes plus the stem-helix S2 epitope) to combat future VOCs and slow the emergence of resistant escape variants in the therapeutic setting (9). Furthermore, from a public health perspective, pan-sarbecovirus bNAbs could serve as the bedrock of pandemic preparedness and biodefense strategies to contain outbreaks and reduce population-level risk in the event of future zoonotic spillovers. For example, individual bNAbs or cocktails could be deployed in a ring-prophylaxis approach to simultaneously curtail transmission and limit disease severity.

Our data also validate mRNA-LNPs as a promising mAb delivery modality. Thus far, the vast majority of clinically utilized mAbs have been administered as recombinant proteins, although this format is limited by the high cost of production and purification (74, 75). The mRNA-LNP platform offers a cost-effective alternative and additionally enables rapid updating by swapping out the antibody variable region sequences in a modular fashion. Administration of mRNA-encoded mAbs has successfully protected against infectious diseases including HIV (34), Chikungunya (76), *Pseudomonas* (77), and SARS-CoV-2 (Fig. 7D) (78, 79) in small animal models, although translation to primates (including humans) has been limited by poor expression (76, 80, 81). Nevertheless, low-level expression may still confer protection if the encoded mAb is exceptionally potent (80), like many of the pan-sarbecovirus bNAbs described here (Fig. 2B). Furthermore, recent advances in LNP engineering have enabled tissue-specific targeting and thus *in situ* expression (78, 82, 83), which may afford protection at lower local mAb concentrations. Another advantage of the mRNA mAb delivery platform is that it preserves the native mAb glycosylation profile, which may affect mAb half-life and effector function (77, 81, 84). Glycosylation of mRNA-delivered mAbs has primarily been studied in the context of IgA Fc PNGSs (77, 81), but should apply to variable region PNGSs like those identified in the heavy chains of Ab537 and Ab570 (Fig. 3A, 3E). While we were unable to identify a functional role for these glycans in RBD recognition and sarbecovirus neutralization, the fact that these PNGS sequons were introduced and selected for over the course of affinity maturation suggests they may be beneficial. Overall, the pan-sarbecovirus bNAbs identified here contribute to pandemic preparedness by informing both vaccine design strategies and therapeutic mAb development.

## Materials and Methods

All materials and methods are described in the *SI Appendix*, including sample collection, pseudovirus neutralization assay, B cell isolation, single-cell BCR amplification, antibody cloning and expression, analysis of antibody mutation probabilities, binding and competition ELISAs, site-specific glycan analysis, protein expression and purification, X-ray crystallography, cryo-EM sample preparation, cryo-EM data collection and processing, modeling of N-linked glycans on Ab537 and Ab570, negative stain electron microscopy, mRNA production, mRNA encapsulation into lipid nanoparticles, quantification of functional Ab401 in mouse serum, generation and titration of viral stocks for *in vivo* challenge, and *in vivo* challenge studies.

## Supplementary Material

Dataset S2

Dataset S1

Supplementary Information

## Data Availability

The crystal structure of Ab401–SARS-CoV-2 RBD was deposited in the PDB (PDB 9ZDU) (58) and the density map for the cryo-EM structure of Ab568-SARS-CoV-2_WA1 spike was deposited in the EMDB (EMD-74077) (60). All monoclonal antibody isolate sequences are available in Dataset S1.

## Significance Statement

Pan-sarbecovirus broadly neutralizing antibodies (bNAbs) are urgently needed to combat SARS-CoV-2 variants, mitigate future zoonotic spillover, and inform vaccine design. Here we describe the isolation of bNAb lineages from a participant with hybrid immunity, which target highly-conserved receptor binding domain epitopes. The five broadest bNAbs potently neutralized all members of a multiclade sarbecovirus panel and exhibited immunogenetic and structural features that make them prime targets for vaccine elicitation. Prophylactic administration of one bNAb as recombinant protein or mRNA protected against an infectious challenge with SARS-CoV-2 and a bat sarbecovirus. These results broaden the armamentarium of antibodies to prevent and treat sarbecovirus infection and establish mRNA-based antibody delivery as a cost-effective alternative to conventional, protein-based approaches.
