# Supplementary material for: mRNA delivery of a class 1/4 SARS-CoV-2 neutralizing antibody protects against diverse sarbecoviruses in a lethal mouse challenge model
Source: Proc Natl Acad Sci U S A. Author manuscript; Available in PMC 2026 Jun 15. (PMC13268366; doi:10.1073/pnas.2536870123)
Supplement: Supplementary Information [file NIHMS2182547-supplement-Supplementary_Information.docx]

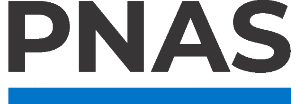


**Supporting Information for**

mRNA delivery of a class 1/4 SARS-CoV-2 neutralizing antibody protects against diverse sarbecoviruses in a lethal mouse challenge model

Ashwin N. Skelly^a,b,1^, Chengcheng Fan^c,1^, Jennifer R. Keeffe^c^, Arya Ökten^d,e^, Edem Gavor^c^, Maddy L. Newby^f^, Joel D. Allen^f^, Edward F. Kreider^a^, Wenge Ding^a,b^, Rebecca A. Osbaldeston^a,b^, Younghoon Park^a,b^, Andrew J. Connell^a,b^, Melinda G. Lituchy^a,b^, Frederic Bibollet-Ruche^a,b^, Katelyn M. Radford^c^, Anthony P. West Jr.^c^, Mario A. Peña-Hernández^d,e^, Kendra Cruickshank^a,b^, Weimin Liu^a,b^, Yingying Li^a,b^, Amie Albertus^a^, Brieyanna McWilliams^a^, Ronnie M. Russell^a,b^, Kylie M. Konrath^g,h^, Jonathan L. Torres^h^, Meng Yuan^h^, Hongmei Gao^i,j^, David C. Montefiori^i,j^, Michael S. Saag^k^, Paul A. Goepfert^k,l,m^, Daniel W. Kulp^g^, Andrew B. Ward^h^, Ian A. Wilson^h^, George M. Shaw^a,b^, Raiees Andrabi^a^, Max Crispin^f^, Drew Weissman^a^, Craig B. Wilen^d,e^, Pamela J. Bjorkman^c^, Beatrice H. Hahn^a,b,2^

^a^Department of Medicine, University of Pennsylvania; Philadelphia, PA 19104, USA.

^b^Department of Microbiology, University of Pennsylvania; Philadelphia, PA 19104, USA.

^c^Division of Biology and Biological Engineering, California Institute of Technology; Pasadena, CA 91125, USA.

^d^Department of Laboratory Medicine, Yale School of Medicine; New Haven, CT 06510, USA.

^e^Department of Immunobiology, Yale School of Medicine; New Haven, CT 06510, USA.

^f^School of Biological Sciences, University of Southampton; Southampton, SO17 1BJ, UK.

^g^Vaccine and Immunotherapy Center, The Wistar Institute; Philadelphia, PA 19104, USA.

^h^Department of Integrative Structural and Computational Biology, The Scripps Research Institute; La Jolla, CA 92037, USA.

^i^Duke Human Vaccine Institute, Duke University Medical Center; Durham, NC 27710, USA.

^j^Department of Surgery, Duke University Medical Center; Durham, NC 27710, USA.

^k^Department of Medicine, University of Alabama at Birmingham; Birmingham, AL 35233, USA.

^l^Department of Microbiology, University of Alabama at Birmingham; Birmingham, AL 35233, USA.

^m^O’Neal Comprehensive Cancer Center, University of Alabama at Birmingham; Birmingham, AL 35233, USA.

^1^A.N.S. and C.F. contributed equally to this work.

^2^To whom correspondence may be addressed. **Email:** bhahn@pennmedicine.upenn.edu

**This PDF file includes:**

Supporting Text

Figures S1 to S10

Tables S1 to S2

Legends for Datasets S1 to S2

SI References

**Other supporting materials for this manuscript include the following:**

Datasets S1 to S2

Supporting Information Text

**Extended Methods**

**Sample collection**

Plasma and peripheral blood mononuclear cell (PBMC) samples from participant CR0011 were collected as previously described (1). Briefly, the University of Alabama at Birmingham COVID-19 convalescent cohort was established in March 2020 at the 1917 Clinic. All participants were enrolled after obtaining written informed consent and approval from the Institutional Review Board (IRB-160125005). Blood samples were collected longitudinally under the appropriate IRB guidelines. Samples were analyzed under code and did not have linkage to patient information.

**Pseudovirus neutralization assay**

Plasma and mAbs were tested for neutralization activity using a lentiviral pseudovirus assay as previously described (1, 2). Briefly, SARS-CoV-2, SARS-CoV-2 VOCs, SARS-CoV, WIV1, Khosta2/SARS-CoV, LYRa3/SARS-CoV, and HKU5 Spikes (containing either a 14, 19, or 21 amino acid cytoplasmic tail truncation) were pseudotyped into an HIV-1 nanoluciferase reporter backbone by co-transfection into HEK 293T cells using FuGENE 6 transfection reagent (Promega E2692). Chimeric Khosta2/SARS-CoV and LYRa3/SARS-CoV Spikes were generated by replacing the RBD of SARS-CoV Spike with the RBD of either Khosta2 or LYRa3 Spike, as previously described (3, 4). Pseudovirus stocks were titrated in human ACE2-expressing HEK 293T clone 22 cells (2). Pseudovirus was incubated with five-fold serial dilutions of mAb (starting at 25 µg/mL) or heat-inactivated plasma (starting at 1:20) for 1 hr at 37 °C and then was used to infect 1.5 × 10^4^ human ACE2-expressing HEK 293T clone 22 cells at a multiplicity of infection (MOI) of 0.3 (2). Following culture for 48 hr at 37 °C with 5% CO_2_, cells were lysed with 0.5% Triton-X 100 in PBS and nanoluciferase activity was determined using Nano-Glo Luciferase Assay per the manufacturer’s instructions (Promega N1150) and quantified on a BioTek Synergy Neo2 plate reader (Agilent). Luminescence in wells with no plasma or mAb was set to 100% and the dilution at which luminescence was reduced to 50% (inhibitory dose 50, ID_50_; inhibitory concentration 50, IC_50_) was calculated as the average of two technical duplicates. Titration and neutralization of merbecovirus HKU5 was performed in BHK-21 cells stably expressing mink ACE2.

**B cell isolation**

Staining and fluorescence-activated cell sorting of antigen-specific B cells was performed as previously described (5), with the following modifications. Cryopreserved PBMCs were thawed, stained with Zombie-NIR viability dye (Biolegend 77184), washed, and stained with a cocktail of antibodies against CD3 (clone OTK3, Invitrogen 47-0037-41), CD8α (clone OKT8, Invitrogen 47-0086-42), CD14 (clone 61D3, Invitrogen 47-0149-42), CD16 (clone eBioCB16, Invitrogen 47-0168-41), CD19 (clone HIB19, Biolegend 302244), IgD (clone IA6-2, Biolegend 348209), IgM (clone G20-127, BD 740595), IgG (clone G18-145, BD 561296), and IgA (polyclonal, Southern Biotech 2050-02). Cells were then washed three times and stained either with SARS-CoV-2_WA1 RBD-His (6) followed by an anti-His antibody (clone J095G46, Biolegend 362603) or with fluorophore-conjugated SARS-CoV-2_WA1 RBD, SARS-CoV RBD, and Pang17 RBD. Single, antigen-specific CD3^-^CD8α^-^CD14^-^CD16^-^CD19^+^IgM^-^IgD^-^IgA^-^IgG^+^ or CD3^-^CD8α^-^CD14^-^CD16^-^CD19^+^IgM^-^IgD^-^IgG^-^IgA^+^ cells were sorted using a BD FACSMelody Cell Sorter into individual wells of a 96-well plate containing lysis buffer. Cell lysates were centrifuged at 300 x g for 5 min at 4 °C and stored at -20 °C.

**Single-cell BCR amplification**

Immunoglobulin heavy (IgH) and light (IgK, IgL) chain genes were amplified as previously described (5, 7). Briefly, RNA was reverse-transcribed using Superscript III reverse transcriptase (Invitrogen 18080044) via incubation at 42 °C for 10 min, 25 °C for 10 min, 50 °C for 1 hr, and then 94 °C for 5 min. Immunoglobulin genes were amplified by two rounds of nested PCR using HotStart Taq polymerase (Qiagen 203207). Primers and cycling conditions were as previously described (7). Amplicons were visualized by gel electrophoresis, Sanger sequenced (Genewiz), and analyzed computationally using IMGT V-QUEST (8).

**Antibody cloning and expression**

Immunoglobulin heavy and light chain variable regions of interest were synthesized and cloned (GenScript) into human IgG1, IgA1, IgK, or IgL expression vectors (pFUSEss-CHIg and pFUSEss-CLIg series, Invivogen) immediately upstream of the respective constant region using EcoRI/NheI (for IgG1 and IgA1), EcoRI/BsiWI (for IgK), and EcoRI/AvrII (for IgL) restriction sites. Recombinant mAbs were expressed as previously described (5). Briefly, paired heavy and light chain plasmids were co-transfected into 293F cells at a 1:2 ratio using ExpiFectamine (Gibco A14525), purified using rProtein A/Protein G Sepharose Gravitrap kit (Cytiva 28985256), concentrated in 30 kDa centrifugal filters (Sigma UFC903024), and buffer exchanged into PBS. Antibody concentration was measured fluorometrically using a Qubit Protein Assay kit (Invitrogen Q33212). *GnTI*^-/-^ 293F cells (Gibco A39240) were used to produce globally glycan-deficient mAbs as indicated. Site-specific mutant mAbs were generated by mutagenizing the parental plasmids using the Q5 Site-Directed Mutagenesis Kit (NEB E0554S) according to the manufacturer’s instructions. Endotoxin-free, recombinant Ab401 for passive immunization studies was produced by GenScript.

**Analysis of antibody mutation probabilities**

The ARMADiLLO computational pipeline was used to analyze amino acid substitution probabilities in immunoglobulin genes (9). For each lineage analyzed, an inferred germline heavy and light chain sequence was generated by reverting all mutations in templated regions to the closest allele in the IMGT database (8, 10). Non-templated nucleotides could not be assessed and as such the non-templated region sequences from the most mature lineage member were used in each case. The default ARMADiLLO parameters were used, except the number of mutations was defined as the number of amino acid substitutions between the iGL and the most mature member of each lineage to enable intra-lineage comparison.

**Binding and competition ELISAs**

Antibody binding to various RBDs was determined by ELISA, performed using a Tecan Evo liquid handling robot as described (11). Nunc MaxiSorp 384-well plates (Millipore Sigma) were adsorbed with 2.5 µg/mL of purified RBD in 0.1 M NaHCO3 pH 9.8 overnight at 4 °C. Plates were blocked with 3% bovine serum albumin (BSA) for one hr at room temperature, followed by incubation for 3 hr at room temperature with four-fold serial dilutions of mAb. Plates were washed with Tris-buffered saline with 0.1% Tween-20 (TBST) and incubated with secondary horseradish peroxidase (HRP)-conjugated goat anti-human IgG (SouthernBiotech) at a 1:100,000 dilution for 1 hr at room temperature, then developed using SuperSignal ELISA Femto Maximum Sensitivity Substrate (Thermo Fisher). Luminescence was measured with a Tecan Infinite M1000 plate reader. ELISA data were collected in duplicate and midpoint binding (EC_50_ values) were obtained with GraphPad Prism 10.6.1 using a four-parameter dose-response curve fit.

Competition ELISAs were performed using a Tecan Evo liquid handling robot as described (11). Nunc MaxiSorp 384-well plates (Millipore Sigma) were adsorbed with 5 µg/mL of Fab targeting a known RBD epitope in 0.1 M NaHCO_3_ pH 9.8 and incubated overnight at 4 °C. After aspiration of the Fab solution, plates were blocked with 3% BSA in TBST for one hr then SARS-2 Wuhan-Hu-1 RBD (5 µg/mL) was added and incubated for 2 hr. RBD was removed via aspiration and 1 µg/mL IgG was added and incubated for 3 hr. Plates were washed with TBST and bound IgG was detected using HRP-conjugated goat anti-human IgG Fc (SouthernBiotech) (1 hr, room temperature) and developed with SuperSignal ELISA Femto Substrate (Thermo Fisher). Luminescence was measured with a Tecan Infinite M1000 plate reader. Measurements were performed in quadruplicate and means are shown as a heat map with red indicating no binding (competition with the Fab) and white indicating binding (no competition with the Fab).

**Site-specific glycan analysis**

50 µg aliquots of each sample were denatured for 1 hr in 50 mM Tris/HCl, pH 8.0 containing 6 M of urea and 5 mM dithiothreitol (DTT). Samples were then reduced and alkylated by adding 20 mM iodoacetamide (IAA) and incubated for 1 hr in the dark, followed by a 1 hr incubation with 20 mM DTT to eliminate residual IAA. The alkylated mAb samples were buffer-exchanged into 50 mM Tris/HCl, pH 8.0 using Vivaspin columns (10 kDa) and were digested overnight using trypsin (Mass Spectrometry Grade, Promega) at a ratio of 1:30 (w/w). The next day, the peptides were dried and extracted using an Oasis HLB µElution Plate (Waters).

The peptides were dried again, resuspended in 0.1% formic acid, and analyzed by nanoLC-ESI MS with a Vanquish Neo (Thermo Fisher) system coupled to an Orbitrap Eclipse Tribrid mass spectrometer (Thermo Fisher) using stepped higher energy collision-induced dissociation (HCD) fragmentation. Peptides were separated using a μPAC Neo HPLC Column (180 µm × 110 cm). A trapping column (PepMap 100 C18 3 μM 75 μM × 2cm) was used in line with the LC prior to separation with the analytical column. The LC conditions were as follows: 180 min linear gradient consisting of 2.4-20% acetonitrile in 0.1% formic acid, then a 10 min linear gradient consisting of 20-32% acetonitrile, followed by 5 min of 76% acetonitrile in 0.1% formic acid. The flow rate was set to 300 nL/min. The spray voltage was set to 2.5 kV and the temperature of the heated capillary was set to 55 °C. The ion transfer tube temperature was set to 275 °C. The scan range was 350−2000 m/z. The stepped HCD collision energies were set to 15, 25, and 45% and the MS2 for each energy was combined. Precursor and fragment detection was performed using an Orbitrap at a resolution MS^1^ = 240,000, MS^2^ = 30,000. A standard AGC target for MS^1^ (4e^5^) and MS^2^ (1e^4^) and auto injection times (MS^1^ = 50 ms; MS^2^ = 54 ms) were used.

Glycopeptide fragmentation data were extracted from the raw file using Byos (Version 5.5; Protein Metrics Inc.). The glycopeptide fragmentation data were evaluated manually for each glycopeptide; the peptide was scored as true-positive when the correct b and y fragment ions were observed along with oxonium ions corresponding to the glycan identified. The MS data was searched using the Protein Metrics 305 N-glycan library. The relative amount of each glycan at each site as well as the unoccupied proportion were determined by comparing the extracted chromatographic areas for different glycotypes with an identical peptide sequence. All charge states for a single glycopeptide were summed. The precursor mass tolerance was set at 4 ppm and 10 ppm for fragments. A 1% false discovery rate (FDR) was applied. The relative amount of each glycan at each site as well as the unoccupied proportion were determined by comparing the extracted ion chromatographic areas for different glycopeptides with an identical peptide sequence. Glycans were categorized according to the composition detected.

HexNAc(2)Hex(9−3) was classified as M9 to M3. Any of these structures containing a fucose were categorized as FM (fucosylated mannose). Complex-type glycans were classified according to the number of HexNAc subunits and the presence or absence of fucose. Core glycans refer to truncated structures smaller than M3. As this fragmentation method does not provide linkage information, compositional isomers are grouped.

**Protein expression and purification**

Proteins used for antigen-specific B cell sorting were produced as follows. Biotinylated SARS-CoV-2_WA1 RBD (GenBank MN985325.1), SARS-CoV RBD (GenBank AAP13441.1), and Pang17 RBD (GenBank QIA48632) were produced by co-expressing the RBD (12) with the biotinylation enzyme BirA (kind gift of Michael Anaya, CalTech) in Expi293F cells (ThermoScientific) as described (13). RBD constructs encoded RBD residues (SARS-CoV-2_WA1: 319-539; SARS-CoV-1: 318-510; Pang17: 317-539) with a 15-residue Avi-tag (GLNDIFEAQKIEWHE) for site-specific biotinylation, followed by a 6xHis tag for purification. Biotinylated RBDs were purified from expression supernatants and stored as described (14).

Proteins used for structural characterization were expressed by transient transfection in Expi293 cells as described (11). His-tagged SARS-CoV-2_WA1 Spike stabilized with HexaPro substitutions (15), 6x-His-tagged SARS-CoV-2_WA1 RBD, and 6x-His-tagged Fabs were first purified by Ni-NTA chromatography (Qiagen). The SARS-CoV-2_WA1 Spike was further purified by size exclusion chromatography (SEC) using a Superose 6 10/300 (Cytiva) column, and the RBD and Fabs were SEC purified using a Superdex 200 10/300 (Cytiva) column. The Spike protein was concentrated to ~6 mg/mL using 100 kDa cutoff concentrators, flash frozen in aliquots, and stored at -80 °C until use. RBD and Fabs were concentrated to 10-15 mg/mL using 10 and 30 kDa cutoff concentrators (EMD Millipore), and the final concentrated proteins were stored at 4 °C.

**X-ray crystallography**

Sitting drop vapor diffusion crystallization trials were set up at room temperature for the Ab401 Fab–SARS-CoV-2 RBD complex using PEG Ion and PEG Rx screen (Hampton Research) by mixing 0.2 µL of protein solution at 15 mg/mL with 0.2 µL of well solution using a TTP LabTech Mosquito instrument at room temperature. Crystals for the Ab401–SARS-CoV-2 RBD complex were observed in several conditions. A final dataset was collected from a crystal grown in PEG Ion condition 43 with 0.2 M ammonium phosphate monobasic and 20% (w/v) polyethylene glycol 3350.

Crystals were cryoprotected in the well solution before flashing-cooling in liquid nitrogen.

X-ray diffraction data were collected at 100K using a wavelength of 1.0358 Å at the Advanced Light Source (ALS) beamline 2.0.1 and a PILATUS pixel array detector (Dectris). The dataset was indexed and integrated with XDS (16) and scaled with Aimless (17). The structure of the Ab401–SARS-CoV-2 RBD complex was solved by molecular replacement using Phaser in Phenix (18). Iterative structural refinement and model building was carried out in Phenix (18) and Coot (19). Crystallographic statistics are reported in **Table S1**.

**Cryo-EM sample preparation**

A SARS-CoV-2 Spike–Fab complex was prepared by incubating purified Spike trimer with Ab568 Fab at a 1.1-fold molar excess per Spike protomer for 30 min at room temperature, yielding a final complex concentration of ~3 mg/mL. Fluorinated octyl maltoside (Anatrace) was added to the sample to a final concentration of 0.02% (w/v) immediately prior to grid preparation as described (20). A 3 µL aliquot of the complex-detergent mixture was applied to a Quantifoil 300-mesh R1.2/1.3 grid (Electron Microscopy Sciences), which had been freshly glow-discharged using a PELCO easiGLOW (Ted Pella) for 1 min at 20 mA. Grids were blotted for 3 s with zero blot force using Whatman No. 1 filter paper at 100% humidity and room temperature and then vitrified in liquid ethane using a Vitrobot Mark IV (Thermo).

**Cryo-EM data collection and processing**

Single-particle cryo-EM datasets for SARS-CoV-2 S-6P in complex with Ab568 Fab were collected using SerialEM (21) automated acquisition on a 200-keV Talos Arctica microscope (Thermo) equipped with a K3 direct electron detector (Gatan). Movies were acquired in super-resolution mode at a calibrated pixel size of 0.4345 Å, using a 3×3 beam-image-shift pattern with three exposures per hole. Each movie consisted of 40 frames, with a total electron dose of 45 e⁻/Å² and a defocus range of -1 to -3 μm. A processing workflow is provided in **fig. S10**. All movies were motion-corrected using patch-based motion correction in cryoSPARC v4 (22) with a 2× binning factor, and contrast transfer function (CTF) parameters were estimated using Patch CTF. Particles were initially picked using the blob picker in cryoSPARC with a diameter range of 100-200 Å, and all picked particles and associated micrographs were manually inspected prior to extraction. Extracted particles were subjected to multiple rounds of 2D classification to remove ice contamination and junk particles. Remaining high-quality particles were used to generate ab initio reconstructions with three initial volumes, followed by heterogeneous refinement. The final map was obtained through iterative homogeneous refinements in cryoSPARC v4.

A model was generated by first docking coordinates of an AlphaFold 3-predicted (23) Ab568 Fab–SARS-CoV-2-RBD complex into the refined cryo-EM density using UCSF Chimera (24). The remaining regions of the SARS-CoV-2 spike trimer were then fit by docking the corresponding coordinates from PDB 7SC1 into the density map.

**Modeling of N-linked glycans on Ab537 and Ab570**

Ab537 and Ab570 are class 1/4 mAbs with YY sequences in their CDRH3s, and models predicted by AlphaFold 3 (23) for their complexes with SARS-CoV-2 RBD were aligned to the Ab401–SARS-CoV-2 RBD complex with root mean square deviations of 0.7 Å between Ab537 and Ab401 over 186 Cα atoms and 0.7 Å between Ab570 and Ab401 over 194 Cα atoms in their V_H_-V_L_ domains. We therefore used the coordinates of the Ab401–SARS-CoV-2 RBD structure to introduce N-glycan coordinates into the Ab537–RBD and Ab570–RBD models. The appropriate heavy chain residues (residue 23 in the Ab537 model and residue 100_A_ in the Ab570 model) were changed to Asn, and N-acetylglucosamine residues were attached to each Asn residue and fit using Coot (19).

**Negative stain electron microscopy**

SARS-CoV-2 (UK Variant B.1.1.7) Spike was complexed with a 3-fold molar excess of Ab33, Ab19, Ab32, or Ab1 Fab for 30 min at room temperature. Complexes were diluted to 0.02 mg/mL in 1X TBS and stained for 90 s with 2% uranyl formate on carbon-coated copper mesh grids. Grids were imaged with the FEI Tecnai Spirit T12 (120 keV) electron microscope with a Tietz TVIPS CMOS (4K x 4K) camera with the Leginon software (25). Images were collected at -1.50 μm defocus at 52,000x magnification resulting in a pixel size of 2.06. The data were stored in the Appion database (26). Single particles were picked with DogPicker software (27) and stacked with a box size of 256 pixels. RELION (28, 29) was used for 2D classes, 3D classifications, and refinements.

**mRNA production**

Plasmids encoding the heavy and light chains of Ab401 with the human IgG1 or kappa constant region, respectively, were synthesized (GenScript) and used as a template for *in vitro* transcription. Nucleoside-modified mRNA products were produced using T7 RNA polymerase (Megascript) with co-transcriptional RNA capping (CleanCap 3OMe, Trilink) and incorporation of N1-methylpseudouridine in place of uridine (Trilink) as previously reported (5, 30, 31). A plasmid-encoded polyadenylation sequence (101 nt) was incorporated to improve mRNA stability within the cell. Contaminating double stranded byproducts were removed with cellulose purification as previously described (31), mRNA size was assessed using gel electrophoresis, and innate immune triggering was evaluated by interferon-alpha release upon transfection of monocyte-derived dendritic cells (32). mRNA was stored at -20 °C.

**mRNA encapsulation into lipid nanoparticles**

mRNA was formulated in the standard vaccine LNP formulations present in the FDA-approved COVID-19 vaccine using a NanoAssemblr Ignite (Cytiva) (33). Lipids (SM102 50%, DSPC 10%, cholesterol 38.5%, and PEG lipid 1.5%) were dissolved in ethanol and mixed with mRNA dissolved in citrate buffer via microfluidic mixing according to the manufacturer’s instructions. mRNAs encoding the Ab401 heavy chain and Ab401 light chain were mixed at a 1:1 molar ratio prior to formulation. Solvent exchange was performed by dilution into a 10% sucrose-PBS solution followed by concentration in 100 kDa centrifugation filtration tubes (Amicon). All mRNA-LNPs underwent quality assessment via dynamic light scatter to ensure particle size < 100 nm and Polydispersity Index (PDI) < 0.2. mRNA concentration was determined and encapsulation efficiency of > 90% was confirmed using Ribogreen according to the manufacturer’s instructions (Thermo). mRNA-LNPs were stored at an approximate concentration of 1 µg/µL at -80 °C prior to use.

**Quantification of functional Ab401 in mouse serum**

Functional Ab401 was detected in the serum of mice via sandwich ELISA. High-binding 96-well plates (Corning 3690) were adsorbed with 2 µg/mL of purified SARS-CoV-2_WA1 RBD in PBS overnight at 4 °C. Plates were washed three times with 0.05% Tween in PBS, blocked with 2% milk in PBS for one hr at room temperature, and then incubated at room temperature for two hr with three-fold serial dilutions of mAb (starting at 5 µg/mL) or serum (starting at 1:100). Plates were washed three times, incubated with secondary HRP-conjugated goat anti-human IgG (Jackson ImmunoResearch 109-035-088) at a 1:5,000 dilution for one hr at room temperature, washed, and then developed using ABTS solution (Invitrogen 002024). Fluorescence at 405 nm was read with a BioTek Synergy Neo2 plate reader (Agilent) and plasma concentration of human Ab401 was quantified by comparison to a standard curve.

**Generation and titration of viral stocks for *in vivo* challenge**

Viral stocks of SARS-CoV-2_WA1 and BANAL-236 were generated as previously described (34, 35). Briefly, Vero E6-ACE2-TMPRSS2 cells (gift of Barney Graham, NIH VRC) were infected at a multiplicity of infection (MOI) of 0.1 for 2-3 days until cytopathic effects were observed to generate a working viral stock. After infection, the supernatant was clarified by centrifugation and passed through a 0.22 μm filter. Filtered virus was aliquoted and stored at -80 °C. Viral titers were measured by plaque assay using Vero-ACE2/TMPRSS2 as described previously (36).

***In vivo* challenge studies**

K18-hACE2 mice were retro-orbitally administered 20 mg/kg recombinant antibody, 30 μg/mouse mRNA-LNPs, or empty LNPs at an equivalent lipid concentration to the mRNA-LNPs. Two days after antibody administration, mice were anesthetized with isoflurane and infected intranasally with 10^6^ plaque-forming units (PFU) of either SARS-CoV-2_WA1 or BANAL-236 diluted in PBS to a final volume of 50 μL. Survival, body condition, and weights of mice were monitored daily for ten consecutive days. To determine efficacy of recombinant IgG, mRNA-LNP, and empty LNP treatment, mice were bled immediately before and two days after antibody treatment by submandibular bleed. Blood was coagulated overnight at 4 °C and was centrifuged at 3300 x g for 10 min the next day. Serum was collected and stored at -80 °C. All *in vivo* experiments utilized littermate controls and were sex-balanced. All procedures and experiments were performed according to protocols approved by the Yale University Institutional Animal Care and Use Committee (IACUC).

**Figures**

**Figure S1. Isolation of RBD-binding B cells from a participant with hybrid immunity. (A)** Neutralization activity of plasma collected from subject CR0011 at the indicated timepoints, expressed as reciprocal ID_50_. Murine leukemia virus (MLV) is used as a negative control. **(B)** Infection, vaccination, and sampling dates of subject CR0011. **(C)** Representative gating strategy for the fluorescence-activated cell sorting used to isolate RBD-binding B cells from Visit 4. Live CD3^-^CD8α^-^CD14^-^CD16^-^CD19^+^IgD^-^IgM^-^IgG/IgA^+^ B cells that were either SARS-CoV-2_WA1-RBD^+^ single-positive or SARS-CoV-2_WA1-RBD^+^ SARS-CoV-RBD^+^ Pang17-RBD^+^ triple-positive were sorted. **(D)** Fraction of IgM^-^IgD^-^ SARS-CoV-2_WA1-RBD^+^ single-positive B cells that express IgG or IgA at the indicated timepoints, as determined by flow cytometry. **(E)** Number of nucleotide (nt) mutations in the V_H_ (left) and V_L_ (right) antibody genes of RBD-specific B cells isolated at the indicated timepoints. Each circle represents an individual antibody sequence and horizontal bars represent the median. Kruskal-Wallis test with Dunn’s test for multiple comparisons was used to determine statistical significance. P**** < 0.0001; ***P < 0.001; *P < 0.1. **(F)** Average number of nucleotide (nt) mutations in the V_H_ (left) and V_L_ (right) antibody genes of lineages that were found at more than one timepoint. Lineages are color-coded as in **Fig. 1C** and **Dataset S1**.

**Figure S2. RBD-specific antibodies isolated from some IgG^+^, but not IgA^+^, B cells cross-neutralize SARS-CoV.** Neutralization activity of 65 IgG (left) and 25 IgA (right) RBD-binding monoclonal antibodies, expressed as IC_50_ in μg/mL. Immunoglobulin subclass and timepoint of isolation are indicated.

**Figure S3. Phylogeny of sarbecovirus RBDs.** Amino acid sequences of the sarbecovirus RBDs used in this study were aligned using Clustal Omega (Geneious v2025.2.2) followed by manual adjustment. A PhyML tree was generated from the alignment using the LG substitution model and branch support was analyzed by bootstrap (Geneious v2025.2.2). Sarbecovirus clade is indicated to the right. Bootstrap support >75% (100 replicates) are shown alongside the branches. Scale bar represents 10% substitutions per site.

**Figure S4. Neutralizing IgA antibodies recognize class 1 and 2 RBD epitopes.** SARS-CoV-2 Spike (UK Variant B.1.1.7) was complexed with Fabs from Ab33, Ab19, Ab32, and Ab1. Spike is shown in grey and Fabs are show in various colors. These antibodies target class 1 or 2 RBD epitopes as shown.

**Figure S5. Immunogenetic and functional characteristics of previously described human-derived pan-sarbecovirus bNAbs.** **(A)** Immunogenetic features of the listed class 1/4 anti-RBD antibodies, as reported in the original studies and verified by IMGT V-QUEST (8) (when nucleotide sequences were available). N.A., not available. **(B)** Neutralization profiles of the indicated antibodies. Neutralization potency is represented as IC_50_ in μg/mL. To enable direct comparison, all data was generated in the current study. All mAbs recognize RBD, except CC25.103 which recognizes the S2 stem helix epitope.

**Figure S6. Neutralization breadth and potency of pan-sarbecovirus bNAb lineage members increase over the course of lineage maturation.** Neutralization profile of early and late members of three class 1/4 bNAb lineages against an 18-virus, multiclade panel. Potency is expressed as IC_50_ in μg/mL. The visit at which antibody was isolated, along with the lineage designation and epitope class, is provided.

**Figure S7. Evolution of bNAb lineage light chains.** Amino acid alignments of pan-sarbecovirus bNAb light chains with earlier lineage members and the closest germline V_K_/V_L_ and J_K_/J_L_ alleles from the IMGT database (8, 10). Somatically mutated residues in the bNAb sequences are color-coded by estimated mutation probability, as determined by ARMADiLLO (9). Non-templated regions are highlighted in light grey and are excluded from analysis. Residues are numbered using the Kabat scheme via ANARCI (37). The timepoint from which each mAb was isolated is listed as visit number (V1 through V5). CDR, complementarity determining region. The corresponding heavy chain alignments are shown in **Fig. 3A**.

**Figure S8. Compositional site-specific glycan analysis of somatically mutated mAbs. (A)** The graphs summarize quantitative mass spectrometric analysis of the glycan population present at individual N-linked glycosylation sites simplified into categories of glycans. The oligomannose-type glycan series (M9 to M5; Man_9_GlcNAc_2_ to Man_5_GlcNAc_2_) is colored green, afucosylated and fucosylated hybrid-type glycans (hybrid and F hybrid) are hatched pink, and complex glycans are grouped according to the number of antennae and presence of core fucosylation and are colored pink. Unoccupancy of an N-linked glycan site is represented in gray. The PNGS sequon is labelled and includes the flanking residues. Pie charts depict the relative percentage abundance of oligomannose-, hybrid-, complex-type glycans, and unoccupied sites. **(B)** Summary of monosaccharide-level glycan substituent modifications at individual N-glycosylation sites in each sample, including fucosylation in red (top), sialylation in purple (middle), and sulfation in yellow (bottom). HCDR, heavy chain complementarity determining region; HFR, heavy chain framework region.

**Figure S9**. **Ab401 epitope alignment across diverse sarbecovirus RBDs.** Ab401 contact residues were determined using PDBePISA and are highlighted in green. Residues are numbered based on the SARS-CoV-2 RBD.

**Figure S10**. **Single-particle cryo-EM workflow.** Schematic illustrating the data-processing procedure for Ab568–SARS-CoV-2_WA1 Spike-6P trimer complex. Representative micrographs, the data-processing pipeline, and the gold standard Fourier shell correlation (GSFSC) curve for the final reconstruction (calculated using the 0.143 threshold) are shown. The final refined map reveals three Ab568 Fabs bound to Spike, with one Fab engaging each protomer in an RBD “up” conformation. The Ab568 heavy chain (HC) and light chain (LC) are shown in dark and light blue, respectively. The S1 and S2 subunits of the spike are labelled in light and dark grey, respectively. The azimuth plot suggests some preferred orientations.

**Tables**

**Table S1. X-ray data collection and refinement statistics.**

| Wavelength (Å) | 1.0358 |
| --- | --- |
| Resolution range (Å) | 45.84 - 2.60 (2.66 - 2.60) |
| Space group | P4_3_2_1_2 |
| Unit cell (a, b, c, α, β, γ) (Å, °) | 83.6 83.6 217.7 90 90 90 |
| Total reflections (no.) | 313,547 (19,915) |
| Unique reflections (no.) | 24,715 (1,693) |
| Multiplicity | 12.7 (11.8) |
| Completeness (%) | 99.7 (97.4) |
| Mean I/sigma (I) | 12.0 (1.1) |
| Wilson B-factor (Å^2^) | 57 |
| R-merge | 0.18 (2.04) |
| R-meas | 0.19 (2.13) |
| R-pim | 0.053 (0.61) |
| CC1/2 | 0.997 (0.372) |
| CC* | 0.999 (0.737) |
| Reflections used in refinement | 24,680 (1,673) |
| Reflections used for R-free | 1,998 (136) |
| R-work | 0.219 (0.372) |
| R-free | 0.254 (0.412) |
| Number of non-hydrogen atoms (no.) | 5,027 |
| macromolecules | 4,952 |
| ligands | 26 |
| solvent | 49 |
| Protein residues (no.) | 640 |
| RMS (bonds) (Å) | 0.002 |
| RMS (angles) (°) | 0.49 |
| Ramachandran favored (%) | 98.0 |
| Ramachandran allowed (%) | 2.0 |
| Ramachandran outliers (%) | 0.00 |
| Rotamer outliers (%) | 0.18 |
| Clashscore | 6.7 |
| Average B-factor (Å^2^) | 62 |
| macromolecules | 62 |
| ligands | 77 |
| solvent | 52 |
| PDB ID | 9ZDU |

**Table S2. Cryo-EM data collection and data processing parameters.**

| Microscope | Talos Arctica |
| --- | --- |
| Voltage (keV) | 200 |
| Camera | Gatan K3 |
| Magnification | 45000x |
| Frames per movie | 40 |
| Recording mode | Counting |
| Dose rate (e-/pixel/s) | 21.234 |
| Total electron dose (e-/Å^2^) | 45 |
| Defocus range (μm) | -1 to-3 |
| Pixel size (Å) (super-resolution) | 0.435 |
| Total no. of movies collected | 3224 |
|  |  |
| EMD Map |  |
| Total no. of final extracted particles | 410,353 |
| Symmetry imposed | C3 |
| Map resolution (Å) | 5.2 |
| FSC threshold | 0.143 |

**Dataset S1 (separate file). Immunogenetic features of RBD-binding B cell receptors.** The immunoglobulin subclass, timepoint of isolation, sort strategy (SARS-CoV-2_WA1 RBD single positive or SARS-CoV-2_WA1, SARS-CoV, Pang17 RBD triple-positive), and gene segment usage and statistics as identified by the IMGT V-QUEST pipeline (8) of B cell receptors from sorted B cells are listed. Lineage groupings were manually assigned based on gene segment usage and CDRH3 length, and color-coding is consistent with **Fig. 1C-D** and **fig. S1F**.

**Dataset S2 (separate file).** **Site-specific relative abundance of exact glycan compositions detected in somatically mutated mAbs.**Glycopeptide analysis reveals the spectrum of glycan compositions detected at each representative PNGS, with relative abundances expressed as percentages of the total extracted ion chromatogram area under the curve (XIC AUC) sum. The exact glycan compositions are classified into broader categories, detailed in the final column, which were used to represent the compositional site-specific glycosylation patterns in **fig. S8**.

**SI References**

1. W. Liu *et al.*, Predictors of Nonseroconversion after SARS-CoV-2 Infection. *Emerg Infect Dis* **27**, 2454–2458 (2021).

2. F. Schmidt *et al.*, Measuring SARS-CoV-2 neutralizing antibody activity using pseudotyped and chimeric viruses. *J Exp Med* **217** (2020).

3. S. N. Seifert *et al.*, An ACE2-dependent Sarbecovirus in Russian bats is resistant to SARS-CoV-2 vaccines. *PLoS Pathog* **18**, e1010828 (2022).

4. A. A. Cohen *et al.*, Mosaic RBD nanoparticles protect against challenge by diverse sarbecoviruses in animal models. *Science* **377**, eabq0839 (2022).

5. A. N. Skelly *et al.*, Induction of broadly neutralizing HIV antibodies by a two-step mechanism informs vaccine design. *Science* 10.1126/science.aec6396, eaec6396 (2026).

6. F. Amanat *et al.*, A serological assay to detect SARS-CoV-2 seroconversion in humans. *Nat Med* **26**, 1033–1036 (2020).

7. T. Tiller *et al.*, Efficient generation of monoclonal antibodies from single human B cells by single cell RT-PCR and expression vector cloning. *J Immunol Methods* **329**, 112–24 (2008).

8. X. Brochet, M. P. Lefranc, V. Giudicelli, IMGT/V-QUEST: the highly customized and integrated system for IG and TR standardized V-J and V-D-J sequence analysis. *Nucleic Acids Res* **36**, W503–8 (2008).

9. J. S. Martin Beem, S. Venkatayogi, B. F. Haynes, K. Wiehe, ARMADiLLO: a web server for analyzing antibody mutation probabilities. *Nucleic Acids Res* **51**, W51–W56 (2023).

10. M. P. Lefranc *et al.*, IMGT, the international ImMunoGeneTics information system. *Nucleic Acids Res* **37**, D1006–12 (2009).

11. C. Fan *et al.*, Cross-reactive sarbecovirus antibodies induced by mosaic RBD nanoparticles. *Proc Natl Acad Sci U S A* **122**, e2501637122 (2025).

12. A. A. Cohen *et al.*, Mosaic nanoparticles elicit cross-reactive immune responses to zoonotic coronaviruses in mice. *Science* **371**, 735–741 (2021).

13. A. A. Cohen *et al.*, Broad anti-sarbecovirus responses elicited by a single administration of mosaic-8 RBD-nanoparticle vaccine prepared using atomic layer deposition. *iScience* **28**, 113649 (2025).

14. A. A. Cohen *et al.*, Mosaic sarbecovirus nanoparticles elicit cross-reactive responses in pre-vaccinated animals. *Cell* **187**, 5554–5571.e19 (2024).

15. C. L. Hsieh *et al.*, Structure-based design of prefusion-stabilized SARS-CoV-2 spikes. *Science* **369**, 1501–1505 (2020).

16. W. Kabsch, XDS. *Acta Crystallogr D Biol Crystallogr* **66**, 125–32 (2010).

17. M. D. Winn *et al.*, Overview of the CCP4 suite and current developments. *Acta Crystallogr D Biol Crystallogr* **67**, 235–42 (2011).

18. D. Liebschner *et al.*, Macromolecular structure determination using X-rays, neutrons and electrons: recent developments in Phenix. *Acta Crystallogr D Struct Biol* **75**, 861–877 (2019).

19. P. Emsley, B. Lohkamp, W. G. Scott, K. Cowtan, Features and development of Coot. *Acta Crystallogr D Biol Crystallogr* **66**, 486–501 (2010).

20. C. O. Barnes *et al.*, SARS-CoV-2 neutralizing antibody structures inform therapeutic strategies. *Nature* **588**, 682–687 (2020).

21. D. N. Mastronarde, Automated electron microscope tomography using robust prediction of specimen movements. *J Struct Biol* **152**, 36–51 (2005).

22. A. Punjani, J. L. Rubinstein, D. J. Fleet, M. A. Brubaker, cryoSPARC: algorithms for rapid unsupervised cryo-EM structure determination. *Nat Methods* **14**, 290–296 (2017).

23. J. Abramson *et al.*, Accurate structure prediction of biomolecular interactions with AlphaFold 3. *Nature* **630**, 493–500 (2024).

24. E. C. Meng *et al.*, UCSF ChimeraX: Tools for structure building and analysis. *Protein Sci* **32**, e4792 (2023).

25. C. Suloway *et al.*, Automated molecular microscopy: the new Leginon system. *J Struct Biol* **151**, 41–60 (2005).

26. G. C. Lander *et al.*, Appion: an integrated, database-driven pipeline to facilitate EM image processing. *J Struct Biol* **166**, 95–102 (2009).

27. N. R. Voss, C. K. Yoshioka, M. Radermacher, C. S. Potter, B. Carragher, DoG Picker and TiltPicker: software tools to facilitate particle selection in single particle electron microscopy. *J Struct Biol* **166**, 205–13 (2009).

28. S. H. Scheres, RELION: implementation of a Bayesian approach to cryo-EM structure determination. *J Struct Biol* **180**, 519–30 (2012).

29. E. F. Pettersen *et al.*, UCSF Chimera--a visualization system for exploratory research and analysis. *J Comput Chem* **25**, 1605–12 (2004).

30. M. G. Alameh *et al.*, Lipid nanoparticles enhance the efficacy of mRNA and protein subunit vaccines by inducing robust T follicular helper cell and humoral responses. *Immunity* **54**, 2877–2892.e7 (2021).

31. M. Baiersdörfer *et al.*, A Facile Method for the Removal of dsRNA Contaminant from In Vitro-Transcribed mRNA. *Mol Ther Nucleic Acids* **15**, 26–35 (2019).

32. K. Karikó, M. Buckstein, H. Ni, D. Weissman, Suppression of RNA recognition by Toll-like receptors: the impact of nucleoside modification and the evolutionary origin of RNA. *Immunity* **23**, 165–75 (2005).

33. S. Omo-Lamai *et al.*, Physicochemical Targeting of Lipid Nanoparticles to the Lungs Induces Clotting: Mechanisms and Solutions. *Adv Mater* **36**, e2312026 (2024).

34. M. A. Peña-Hernández *et al.*, SARS-CoV-2-related bat viruses evade human intrinsic immunity but lack efficient transmission capacity. *Nat Microbiol* **9**, 2038–2050 (2024).

35. T. Thi Nhu Thao *et al.*, Rapid reconstruction of SARS-CoV-2 using a synthetic genomics platform. *Nature* **582**, 561–565 (2020).

36. J. Wei *et al.*, Pharmacological disruption of mSWI/SNF complex activity restricts SARS-CoV-2 infection. *Nat Genet* **55**, 471–483 (2023).

37. J. Dunbar *et al.*, SAbPred: a structure-based antibody prediction server. *Nucleic Acids Res* **44**, W474–8 (2016).
